# Pristimerin Exacerbates Cellular Injury in Conditionally Reprogrammed Patient-Derived Lung Adenocarcinoma Cells by Aggravating Mitochondrial Impairment and Endoplasmic Reticulum Stress through EphB4/CDC42/N-WASP Signaling

**DOI:** 10.1155/2020/7409853

**Published:** 2020-07-10

**Authors:** Yubo Tang, Yiyan Lei, Shuai Huang, Zhangyan Li, Xiangtian Chen, Honghe Luo, Chao Cheng, Jie Chen, Xuenong Zou, Xiao Chen

**Affiliations:** ^1^Department of Pharmacy, The First Affiliated Hospital, Sun Yat-sen University, 510080 Guangzhou, China; ^2^Department of Thoracic Surgery, The First Affiliated Hospital, Sun Yat-sen University, 510080 Guangzhou, China; ^3^Department of Orthopaedic Surgery, The Second Affiliated Hospital, Guangzhou Medical University, 510260 Guangzhou, China; ^4^Department of Pharmacy, The Boai Hospital of Zhongshan City, 528400 Zhongshan, China; ^5^Department of Orthopaedic Surgery, The First Affiliated Hospital, Sun Yat-sen University, 510080 Guangzhou, China; ^6^Guangdong Provincial Key Laboratory of Orthopedics and Traumatology, The First Affiliated Hospital, Sun Yat-sen University, 510080 Guangzhou, China

## Abstract

Lung cancer is the most common and lethal malignant disease for which the development of efficacious chemotherapeutic agents remains an urgent need. Pristimerin (PRIS), a natural bioactive component isolated from various plant species in the Celastraceae and Hippocrateaceae families, has been reported to exhibit outstanding antitumor effects in several types of cells. However, the underlying mechanisms involved remain poorly understood. Here, we reported the novel finding that PRIS significantly suppressed lung cancer growth in conditionally reprogrammed patient-derived lung adenocarcinoma cells (CRLCs). We demonstrated that PRIS inhibited the cell viabilities, migrative and invaded abilities, and capillary structure formation of CRLCs. Furthermore, our results clarified that PRIS induced mitochondrial dysfunction through reactive oxygen species (ROS) generation, activation of caspase-9, caspase-3, and caspase-4, and expression of endoplasmic reticulum (ER) stress-associated proteins. Inhibition of ER stress by 4-PBA (4-phenylbutyric acid, a specific ER stress inhibitor) or CHOP siRNA transfection ameliorated PRIS-induced loss of mitochondrial membrane potential and intrinsic apoptosis. The present study also provides mechanistic evidence that PRIS suppressed the EphB4/CDC42/N-WASP signaling pathway, which is required for mitochondrial-mediated intrinsic apoptosis, activation of ER stress, and stimulation of caspase-4 induced by PRIS, and consequently resulting in suppressed cell viability, migration, and angiogenesis in CRLCs. Taken together, by providing a mechanistic insight into the modulation of ER stress-induced cell death in CRLCs by PRIS, we suggest that PRIS has a strong potential of being a new antitumor therapeutic agent with applications in the fields of human lung adenocarcinoma.

## 1. Introduction

As the leading cause of cancer mortality with the most common incidence, lung cancer is still therapeutically challenged all over the world [1]. In the past three decades, strategies based on the combination of surgery and chemotherapy regimens have been developed in an initial treatment of lung cancer. However, the overall survival rate for lung cancer has not significantly improved because these tumors have a high incidence of recurrence and commonly lead to death within less than a year from diagnosis. Therefore, extensive research has been done to identify more effectual antitumor regimens.

Pristimerin (PRIS) is a natural quinonemethide triterpenoid compound isolated from various plant species in the Celastraceae and Hippocrateaceae families [2]. PRIS has been reported to possess a variety of pharmacological activities including anti-inflammatory, antiperoxidation, antioxidant, and antimalarial activities [3, 4]. Additionally, PRIS was showed to inhibit tumor growth of various human cancers such as colon [5], prostate [6], pancreatic [7], cervical [8], and multiple myeloma tumors [9]. Although proteasome inhibition, reactive oxygen species (ROS) generation, and endoplasmic reticulum (ER) stress have been implicated in PRIS-induced cell death, the molecular pathways underlying the anticancer effect of PRIS are dependent on the cellular contexts and thus remain to be further investigated [9–11].

Many factors can contribute to the induction of the ER stress and the unfolded protein response (UPR) including overexpression of proteins beyond the capacity of the ER to correctly fold them, inhibition of glycosylation [12], and oxidative stress among others. While moderate ER stress triggers cell survival signaling, severe stress may potentiate cell death [13, 14]. Mitochondrial dysfunction, ROS accumulation, and cytosolic Ca^2+^ increase crosstalk each other and these factors might play some roles in regulating ER stress-associated apoptotic cell death [15]. Overexpression of the transcription factor CHOP participates in ER stress-induced apoptosis, and cells lacking CHOP are protected from apoptosis [16]. It is reported that the induction of ER stress by chemotherapeutic drug could further promote cell death by various mechanisms in cancer cells [17, 18]. Considering that ER stress plays a crucial role in the regulation of cell death, as well as programmed necrosis [19, 20], we speculated that PRIS might induce ER stress-mediated cell death in lung cancer.

For three decades, the mainstay of preclinical cancer therapeutic research has been the use of human cancer cell lines cultured *in vitro* and of xenografts derived from these cell lines grown *in vivo* in immunodeficient mice. Some reports suggested that when the molecular profiles of patient tumors are compared to established cell lines, there is substantial genetic divergence between primary lung cancers and cell lines [21, 22]. The complex heterogeneity of primary tumors lacking in these cell lines prevents the use of such cultures for predicting tumor cell responses and results in barriers to the successful translation of new cancer therapeutics [22]. Establishment and maintenance of long-term *ex vivo* cultures directly from patient-derived tumor tissue samples have been very challenging, but this is starting to change due to recent breakthroughs in two-dimensional (2D) and 3D cell culture technologies. These new primary culture technologies consist of either conditionally reprogrammed (CR) cells cocultured as monolayers (in 2D) with feeder cells (irradiated-3T3 mouse fibroblasts) in the presence of a Rho-associated protein kinase inhibitor (ROCKi) or of patient-derived spheroids or organoids (in 3D) [23–25]. Importantly, these models are not established through xenografting or exogenous gene transfer and they could become critically important in understanding the disease and identifying new therapeutic agents for various types of cancers [26].

In the present study, we generated patient-derived CR cells from lung adenocarcinoma tissues (CRLCs). We characterized these CRLCs with genomic and protein expression profiling in this study and further examined whether PRIS induces CRLCs death via the ER stress pathway and investigated the underlying mechanism. Our results demonstrate that PRIS-induced cell death predominantly occurs through mitochondrial impairment and ER stress via the EphB4/CDC42/N-WASP signaling pathway.

## 2. Materials and Methods

### 2.1. Tissue Processing and Establishment of CRLCs

Lung tissue samples were collected from 12 patients undergoing partial resection of the lung or ultrasound-guided needle biopsy at the First Affiliated Hospital of Sun Yat-sen University. Consent for tissue sample collection was obtained from these patients with nonmetastatic lung adenocarcinoma (age range 47-71 years, mean age 62.2 years). All procedures were performed in accordance with the guidance and approval of the local institutional review board (approval no. 2018153). Fragments of freshly obtained tumor tissues were dissociated using collagenase/hyaluronidase and Dispase (StemCell Technologies, Vancouver, Canada) at 37°C for 3 h with occasional shaking as per the manufacturer's protocol. Once the primary cells were isolated from the parental tissue with collagenase treatment, they were cocultivated with irradiated 3T3-J2 fibroblast feeder cells in full medium [27]. The cell culture medium included complete DMEM with freshly added supplements: 10% fetal bovine serum (FBS), penicillin, streptomycin and glutamine, F12 nutrient mix (all at 1x), 25 *μ*g/mL hydrocortisone, 125 ng/mL epidermal growth factor (EGF), 5 *μ*g/mL insulin, 250 *μ*g/mL fungizone, 10 *μ*g/mL gentamycin, 10 *μ*g/mL nystatin, 0.1 nM cholera toxin, and 10 *μ*M Rho kinase inhibitor Y27632 (Enzo, Catalog No. 270-333M025) [27]. The medium was replaced every 3 d. Differential trypsinization was used to separate the feeder cells from the CRLCs during passaging and seeding of the cells for the experiments [27]. All experiments and characterizations including phenotypic quantification, molecular profiling, and drug testing were done at the same time point, approximately 3-5 weeks after the initiation of the cultivation. CRLCs isolated from the 12 patients would be used for the following experiments.

### 2.2. Immunocytochemistry and Immunofluorescence Labeling

For immunocytochemistry assay, after 15 min fixation in 4% paraformaldehyde, CRLCs were permeabilized for 10 min with PBS containing 0.2% bovine serum albumin (BSA) and 0.1% TritonX-100. Then, the slides were washed and incubated with 3% BSA for 1 h. CK7, CEA, and TTF-1 antibodies were visualized using 3,3′-diaminobenzidine (DAB) chromogen, counterstained with hematoxylin (Mayers hemalum solution, Merck KGaA, Darmstadt, Germany), and mounted with ProLong Gold antifade reagent (Molecular Probes, Paisley, UK) or Pertex (HistoLab, Gothenburg, Sweden).

For immunofluorescence labeling, cells were fixed with 4% paraformaldehyde and nonspecific antibody binding was blocked by incubation in PBS with 5% normal goat serum. Then, cells were incubated with the primary antibody and the DyLight® 488-conjugated or DyLight® 594-conjugated secondary antibody (Abcam, MA, USA). Finally, these cells were stained with Hoechst 33342 or DAPI at room temperature (RT) for 10 min. The results were visualized using a Leica fluorescence microscope (Leica, Wetzlar, Germany) with fluorescein filter set at 350 nm (blue), 488 nm (green), or 590 nm (red).

### 2.3. STR Analysis

Short tandem repeat (STR) analysis (i.e., DNA fingerprinting) was performed using a commercially available kit (Cell ID System; Promega Corporation, Madison, WI). This system allowed the coamplification and three-color detection of 9 loci (8 STR loci and the Y-chromosome-specific Amelogenin). The following STR markers were tested in addition to the Amelogenin locus: CSF1PO, D13S317, D16S539, D5S818, D7S820, THO1, TPOX, and vWA. The PCR amplification was performed according to the manufacturer's recommended protocol and as previously described [28]. Detection of the amplified fragments was achieved with the ABI 3100 genetic analyzer (Applied Biosystems). Data analysis and allele size determination were performed using GeneMapper Software (Applied Biosystems).

### 2.4. PRIS Administration

PRIS powder (Catalog No. P0020) was purchased from Sigma. A 50 mmol/L solution was prepared by mixing powder dissolved in sterile dimethyl sulfoxide (DMSO) and then aliquoted and stored at -20°C. The amount of DMSO added to the cell culture was less than 0.8% in all cases.

### 2.5. Cell Proliferation Assay

CRLCs were treated with PRIS for 24, 48, and 72 h, and cell numbers were determined using the MTS assay (CellTiter 96 Aqueous One Solution Cell Proliferation Assay, Promega, Madison, WI, USA) according to the manufacturer's instructions. The absorbance was measured at 490 nm using a spectrophotometer. All experiments were repeated and read at least three times for each concentration.

### 2.6. LDH Release Assay

CRLCs were seeded into 96-well plates at a cell density of 8 × 10^3^ cells/well, cultivated overnight, and treated with PRIS diluted in F-medium for 24 h. Lactate dehydrogenase(LDH) release was examined using a CytoTox 96® Nonradioactive Cytotoxicity Assay kit (Promega, Madison, WI, USA) in accordance with the manufacturer's instructions. Colorimetric absorbance was measured at 450 nm with a microplate reader. The release of intracellular LDH into the extracellular medium was measured by determining the enzyme activity and was expressed according to the following formula: Percentage of cytotoxicity = (Absorbance of experimental samples/Absorbance of maximun LDH release) × 100%.

### 2.7. Calcein-AM/Ethidium Homodimer-1 Cell-Survival (Live-Dead) Assay

Cell viability was determined using a calcein-AM/ethidium homodimer-1 (EthD-1) dual-staining assay kit (Molecular Probes Inc., Eugene, OR, USA) as previously described [29]. The CRLCs were treated with or without 2, 4, or 8 *μ*M PRIS for 24 h. After treatment, the culture medium was removed and cells were rinsed with warm PBS very gently as not to stir cells. Subsequently, 2 *μ*M calcein-AM and 4 *μ*M EthD-1 in 100 *μ*L PBS were added to each culture well and incubated at 37°C for 30 min. The number of live (green) and dead (red) cells was determined with a fluorescence microscope (Leica, Wetzlar, Germany).

### 2.8. Colony Formation Assay

CRCs were seeded on 6-well plates at a density of 3000 cells per plate. Cells were then treated with DMSO or PRIS (1, 2, 4, 8, or 16 *μ*M) for 24 h. The cells were maintained at 37°C and allowed to grow for 3 weeks. Colonies of cells were then fixed with cold methanol for 20 min and stained with 0.1% crystal violet [30].

### 2.9. Apoptosis Assay by Flow Cytometry Analysis

CRLCs were seeded in six-well plates and allowed to attach for 24 h. After transfection with caspase-4 siRNA for 48 h, CRLCs were treated by PRIS for 24 h, and cell apoptosis was assessed by Annexin V/PI apoptosis detection kit following the manufacturer's instructions (Miltenyi, Bergisch Gladbach, Germany). Briefly, the cells were gently trypsinized, washed with PBS, resuspended in binding buffer, and incubated with Annexin V-FITC and PI at RT for 10 min. Flow cytometric analysis was performed using a LSRII system (BD™, Heidelberg, Germany) and FlowJo software (Tree Star, Inc., Ashland, OR, USA).

### 2.10. Migration and Invasion Assay

Cell migration and invasion were evaluated using Transwell insert chambers with an 8 *μ*m pore size (Corning, Maine, USA) coated with or without Matrigel (BD Biosciences, Bedford, MA), and 5 × 10^4^ cells were seeded in the serum-free medium in the upper chamber. The lower chamber was filled with 600 *μ*L DMEM with 10% FBS. After 48 h, the cells that had traversed the membrane were fixed, stained in Alexa Fluor 488® phalloidin solution for 30 min, and counted [31].

### 2.11. Capillary Tube Formation Assay

*In vitro* angiogenesis assay was performed using BD Bio-Coat Angiogenesis System according to the manufacturer's instructions. Briefly, 1 × 10^5^ cells/mL primary human umbilical vein endothelial cells (HUVECs) (C-12200, PromoCell, Germany) was seeded onto the Matrigel-precoated well present with or without conditioned medium from PRIS-treated (24 h) CRLCs. Tube formation was assessed after 18 h, and photographic imaging was performed under an inverted light microscope (Zeiss Axio Observer Z1, Germany).

### 2.12. Measurement of ROS Production

The levels of ROS induced by PRIS in CRLCs were measured using 2,7-dichlorodihydro-fluorescindiacetate (DCFH-DA, Sigma-Aldrich, St. Louis, USA) as a fluorescent probe as described previously [28]. Cells were washed twice with PBS, incubated with DCFH-DA (10 *μ*M) for 30 min at 37°C, and again washed three times with PBS. The intensity of 2′,7′-dichlorofluorescin (DCF) fluorescence was evaluated using a fluorescence microplate reader with excitation and emission wavelengths at 488 and 520 nm, respectively.

### 2.13. Assessment of Mitochondrial Membrane Potential

To assess mitochondrial membrane potential (MMP) loss, cells were treated with PRIS for the indicated times. Cells were washed twice with PBS, resuspended in PBS containing 20 nM DiOC_6_ and 20 *μ*g/mL PI, and then incubated at 37°C for 15 min. Fluorescence intensity was examined in cells at channel FL1 for DiOC_6_ or channel FL3 for PI. Nonapoptotic cells were stained green with DiOC_6_ and apoptotic cells showed decreased intensity of DiOC_6_ staining, while necrotic cells were stained red with PI. Fluorescence intensity was then measured by flow cytometry using excitation and emission wavelengths of 482 and 504 nm, respectively [32]. At least 20,000 events were analyzed per sample and each sample was performed in duplicate.

### 2.14. Measurements of Ratio of Glutathione (GSH) and Glutathione Disulfide (GSSG)

GSH/GSSG ratio quantification was conducted using the Promega GSH/GSSG-Glo™ Assay (Promega, Madison, WI). Cells were plated at a concentration of 3 × 10^3^ cells/well in 96-well plates. After indicated duration of treatment, media was removed and the assay was conducted according to the manufacturer's instructions. Luminescence was measured using the Synergy H1 Multimode reader. GSH/GSSG ratios were calculated using the following equation: GSH/GSSG = [Total GSH − (2 × GSSG)]/GSSG.

### 2.15. Caspase Activity Assay

CRLCs with or without PRIS (2, 4, and 8 *μ*M) were harvested and suspended in lysis buffer and incubated on ice for 1 h. The cell lysates were centrifuged at 4°C (12,000 × g, 30 min). After centrifugation, supernatants were collected, and the protein concentration was measured immediately using a BCA assay kit (in accordance with the manufacturer's instructions). For the caspase activity assay, the cell lysates were placed in 96-well plates and incubated with the specific caspase substrates (Ac-LEHD-AMC for caspase-9, Ac-DEVD-AMC for caspase-3, and Ac-LEVD-AFC for caspase-4) at 37°C for 1 h. The activity was determined using the fluorimeter (excitation/emission 380/440 nm for AMC and 400/500 nm for AFC) [33].

### 2.16. Transfection with Small Interfering RNA (siRNA)

Cells were seeded in 35 mm dishes at a density of 5 × 10^5^ cells/dish and cultured without antibiotics. After overnight culture, CRLCs were transfected with siRNA targeting caspase-4, CHOP, EphB4, N-WASP, or scrambled control siRNA using the Lipofectamine RNAiMax reagent (Invitrogen, Carlsbad, CA, USA) according to the manufacturer's instructions. Cellular levels of the proteins specific for the siRNA transfection were checked by quantitative real-time RT-PCR (qRT-PCR) and Western blot, and all experiments were performed 48 h after transfection.

### 2.17. RNA Extraction and qRT-PCR

Total RNA was isolated by using RNeasy Mini Kit (QIAGEN, Hilden, Germany). Total RNA (300 ng) from each sample was subjected to reverse transcription using a cDNA reverse transcription kit (Applied Biosystems, Foster City, CA, USA) according to the manufacturer's protocol. qRT-PCR amplifications were performed using the SYBR Premix Ex Taq™ II kit (Applied Biosystems, Foster City, CA, USA) by CFX96™ real-time PCR detection system. A total volume of 25 *μ*L with the condition (95°C, 30 s for initial denaturation, 40 cycles of 95°C for 5 s and 60°C for 30 s in addition with a Melt curve reaction) was performed. Data were normalized to GAPDH expression, and relative expression was calculated by the 2^−*ΔΔ*CT^ method.

### 2.18. Western Blot Analysis

Protein extraction and Western blot were done as described previously [29]. Samples containing equal amounts of protein (30 *μ*g) were separated by electrophoresis on polyacrylamide SDS gels and transferred to polyvinylidene difluoride (PVDF, Amersham, UK) membranes by electroblotting. Subsequently, the membranes were saturated with 5% (*v*/*v*) FBS and probed with primary antibodies overnight at 4°C. Membranes were then extensively washed and probed with horseradish peroxidase-linked secondary antibody (Cell Signaling Technologies, Beverly, MA, USA), followed by enhanced chemiluminescence (GE Healthcare, Buckinghamshire, UK) detection. Membranes were stripped and reprobed for total protein, GAPDH, or *α*-tubulin to demonstrate equal loading. The values of band intensities were quantified by Quantity One 4.6.2 software (Bio-Rad Laboratories, Hercules, CA, USA) to the respective protein loading controls. All immunoblots shown here are representatives of at least three independent experiments.

### 2.19. Statistical Analysis

Numerical data are presented as the means ± standard deviation from at least three individual experiments with cells from different donors, unless otherwise indicated. Statistical comparisons between groups were performed by one-way ANOVA followed by Student's *t*-test using SPSS 16.0 software package. Statistical significance was established at ∗*P* < 0.05 and ∗∗*P* < 0.01 versus indicated group.

## 3. Results

### 3.1. Growth State and Molecular Characteristics of the CRLCs

**Figure 1:**
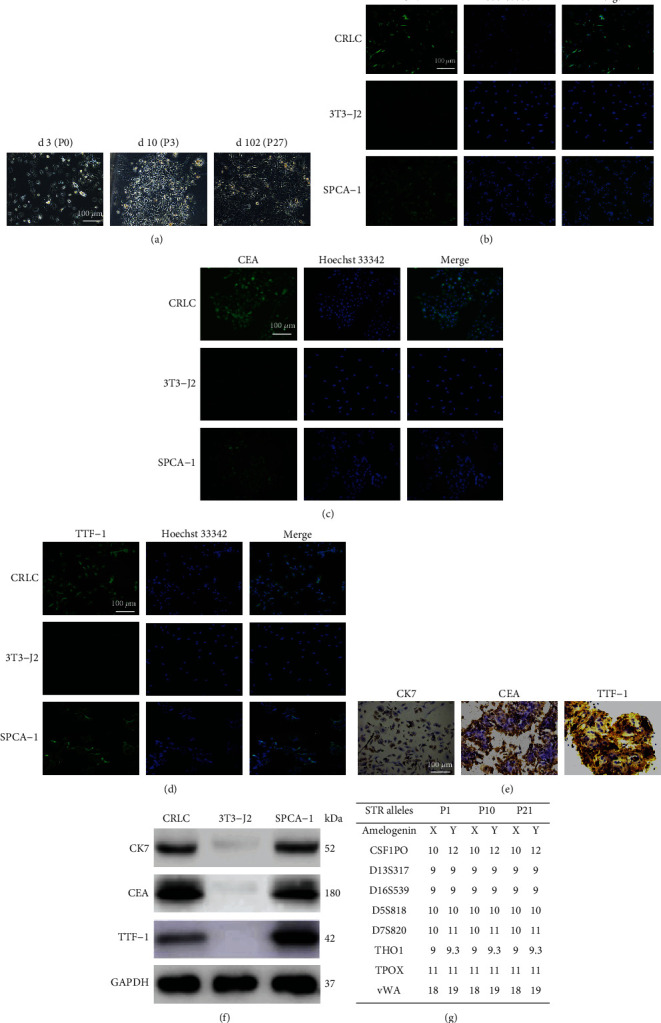
Characterization of conditionally reprogrammed lung cancer cells (CRLCs). (a) The morphology of CRLCs in different growth periods. (b–d) Representative immunofluorescence images of CRLCs, 3T3-J2, and a lung adenocarcinoma cell line SPCA-1 showing phenotypic classifiers for CK7, CEA, and TTF-1. The nuclei were counterstained with Hoechst 33342 for DNA. (e) Immunocytochemistry staining of CRLCs, 3T3-J2, and SPCA-1 with the indicated markers CK7, CEA, and TTF-1. (f) Western blot analysis of CRLCs, 3T3-J2, and SPCA-1 with the following markers: CK7, CEA, and TTF-1. (g) STR analysis of CRLCs. All Western blot band intensities were normalized to GAPDH.

A small quantity of adherent cells could be easily observed following the first 3 d of isolation. As the culture time prolonged, the number of attached cells increased and the cells, appearing fusiform or polygon, gradually gathered into cluster or scattered over the bottom of the bottle. After 4-5 d, they entered into the rapid growth period and cell passage was usually performed every 3-4 d culture. The cells had a strong proliferative ability even after continuous culture *in vitro* for 3 months and more than 20 passages (Figure 1(a)).

To define and quantify the phenotypic variation in the CRLCs, we performed immunofluorescence labeling of the cells. The results revealed that after 10 times of passage, the *in vitro* cultured cells were highly purified. The cells were positive for cytokeratin (CK) 7, CEA, and TTF-1, the biomarkers for epithelial-derived tissue and non-small-cell lung cancer (Figures 1(b)–1(d)), while 3T3-J2 cells or SPCA-1 (a lung adenocarcinoma cell line) cells were negative or positive for all these three biomarkers. We confirmed the expression of these cell type-specific markers using immunocytochemistry and Western blot from the corresponding CRLCs, 3T3-J2, and SPCA-1 cells (Figures 1(e) and 1(f)).

To verify that our immortalized CRLC cultures were not contaminated with another cell line during prolonged passaging, we performed DNA fingerprinting analysis at nine STR loci and at the Y-specific Amelogenin locus [26]. The data in Figure 1(g) demonstrated that the CRLC cultures, when analyzed at early, middle, and late passages, are identical at all 9 loci, thus confirming their identity.

### 3.2. PRIS Inhibits Proliferation and Colony Formation Ability of CRLCs

**Figure 2:**
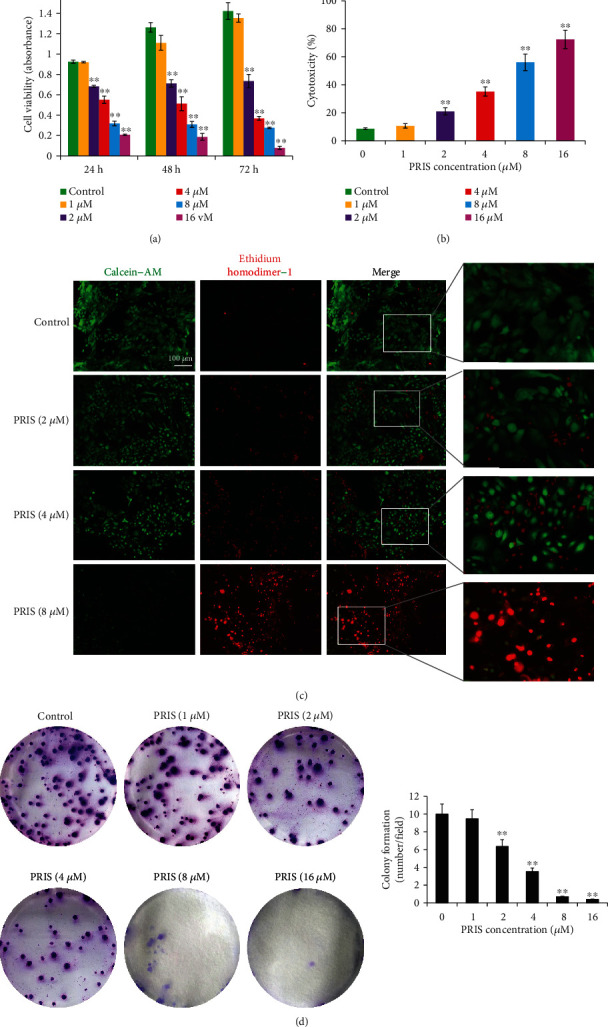
Effect of PRIS on cell proliferation and colony formation ability in CRLCs. (a) Cells were treated with 0 (control), 1, 2, 4, 8, and 16 *μ*M PRIS for 24, 48, and 72 h followed by MTS assay to determine cell number/cell proliferation, with cells that received no treatment as the negative control (*n* = 6). (b) Cells were treated with the same concentrations as in (a) for 24 h, and cell death was evaluated by LDH assay (*n* = 5). (c) Cell survival was monitored using a calcein-AM/EthD-1 double staining. Micrographs representing cells stained with calcein-AM and EthD-1 after treating the cells for indicated doses for 24 h. Viable cell population appears green (calcein-AM) whereas nonviable/dead cells appear as red (EthD-1) in the fluorescent micrographs. (d) CRLCs were treated with PRIS (0, 1, 2, 4, 8, and 16 *μ*M) for 24 h and then, after withdrawal of the treatment, were left to grow for 3 weeks. A colony formation assay was performed to evaluate the survival of colony-forming cells (number of colonies) (*n* = 6). Data are presented as mean ± SD, ∗∗*P* < 0.01.

In the present study, we first determined the effect of PRIS on CRLC proliferation after treating the cells at various concentrations (0, 1, 2, 4, 8, and 16 *μ*M) for 24, 48, or 72 h. Compared with that of the solvent control (DMSO), treatment with PRIS for all the three time points significantly affected the viability of CRLCs at a concentration of more than 2 *μ*M, while a concentration of 1 *μ*M had no influence on cell proliferation (Figure 2(a)). This concentration range was used in all subsequent experiments. The cytotoxic effects of PRIS were confirmed by LDH assay. As illustrated in Figure 2(b), cells treated with 2 *μ*M PRIS for 24 h showed an increased percentage in LDH release as compared to control cells (8.64 ± 0.65% vs. 21.06 ± 2.57%). Pretreatment with 4, 8, and 16 *μ*M PRIS for 24 h demonstrated even stronger cytotoxic effects as observed by an increase in LDH release to 35.15 ± 2.23%, 56.06 ± 5.85%, and 72.39 ± 6.56%, respectively (*P* < 0.01).

To examine the cytotoxic effects of PRIS, CRLCs were double-stained with calcein-AM/EthD-1 and evaluated qualitatively. While in the absence of PRIS nearly all cells were alive, the addition of 2 *μ*M PRIS induced a significant level of cell damage, as shown by contraction and nuclear membrane shrinkage. More serious cellular injury triggered by PRIS was observed at higher dosages of 4 or 8 *μ*M (Figure 2(c)).

Colony formation assays showed PRIS remarkably decreased colony numbers formed by CRLCs, as compared with the control (Figure 2(d)). This suggested that PRIS suppresses malignant transformation and decreases the tumorigenic potential of the CRLCs.

### 3.3. PRIS Suppresses Migrative and Invasive Ability and Inhibits the Tube Formation of CRLCs

**Figure 3:**
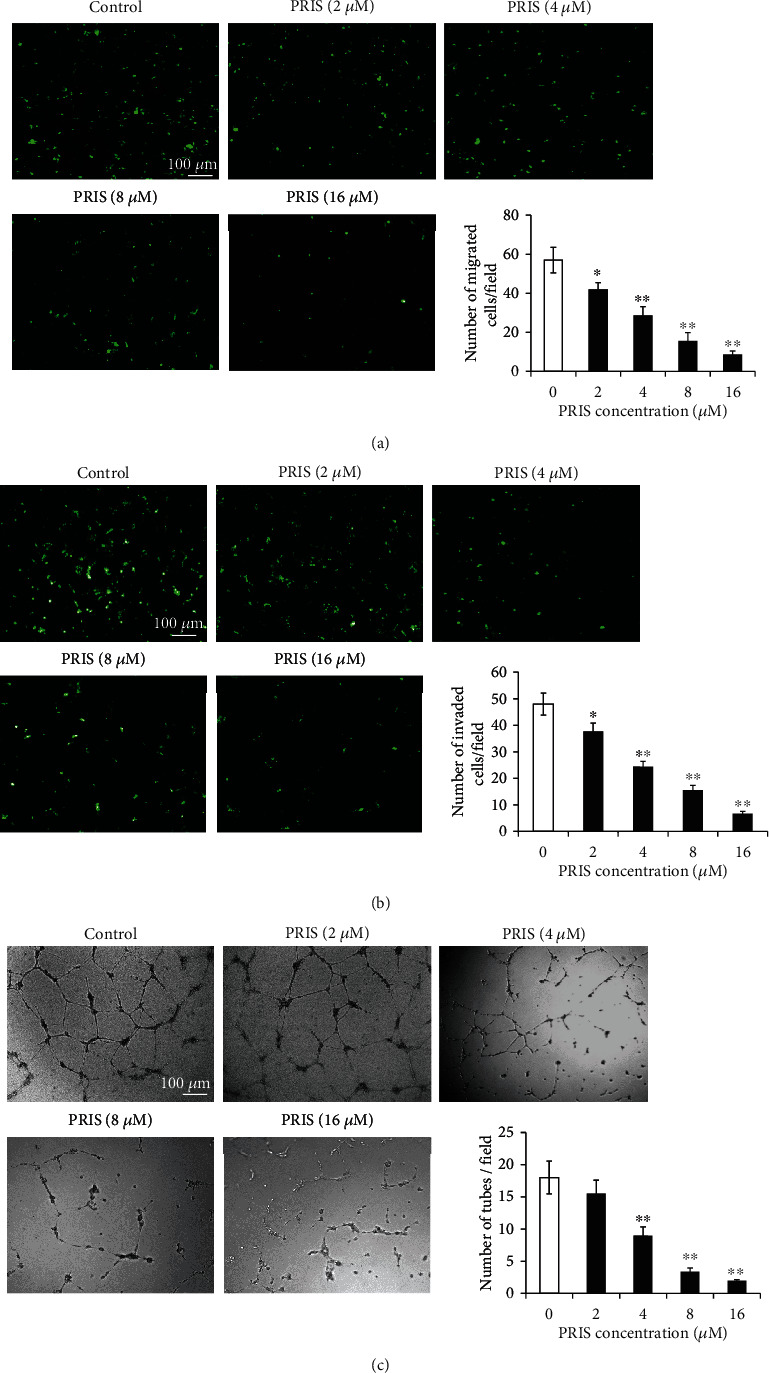
Effect of PRIS on cell migration, invasion, and capillary tube formation ability. (a) Chemotactic movement assessed in a Transwell chamber assay with cells seeded in serum-free medium in the upper chamber, with 10% FBS as chemoattractant in the lower chamber. After 48 h incubation, cells on the upper side of the membrane were removed with a cotton swab, insert membrane was stained with Alexa Fluor 488® phalloidin, and the migrated cells were examined using a fluorescence microscope. The number of migrated cells was quantified by performing cell counts of 10 random fields at ×100 magnification (*n* = 5). (b) The upper chambers were coated with 1 : 8 diluted Matrigel Matrix for 1 h at 37°C; 5 × 10^4^ cells were seeded in the upper chamber with medium containing 10% FBS in the lower chamber. The following procedures were similar to the migration assay as described above in (a) (*n* = 6). (c) After incubating with conditioned medium from CRLCs for 24 h, HUVECs were grown on Matrigel™ for 18 h under normal growth conditions. Capillary tube formation was observed under an inverted light microscope. Five independent fields were assessed for each well, and the average number of tubes/40x field was determined (*n* = 6). Data are presented as mean ± SD, ∗*P* < 0.05, ∗∗*P* < 0.01.

To further confirm the ability of PRIS to decrease CRLC migration and invasion, we evaluated the cell migration and invasion using Transwell plates. The results showed that the number of PRIS-treated cells migrating or invading the lower chamber was much less than that of the control cells (treated with the solvent DMSO). Such a significant reduction was concentration dependent after treatment with 2, 4, 8, or 16 *μ*M of PRIS (Figures 3(a) and 3(b)). Taken together, these findings showed that PRIS decreases CRLC migration and invasion in a dose-dependent manner.

Furthermore, we also checked whether PRIS can regulate angiogenesis in CRLCs. After pretreatment with conditioned medium for 24 h, capillary tube formation assays were performed *in vitro*. After 18 h incubation on Matrigel™, 4 *μ*M PRIS significantly inhibited the number of sprouting tubules by 54.4 ± 11.4% as compared with untreated cells. In the group incubated with 8 and 16 *μ*M PRIS, the majority of the cells remained in individual clusters with lower tubular formation potential (Figure 3(c)).

### 3.4. PRIS Induces Caspase-Dependent Intrinsic Apoptotic Cell Death in CRLCs

**Figure 4:**
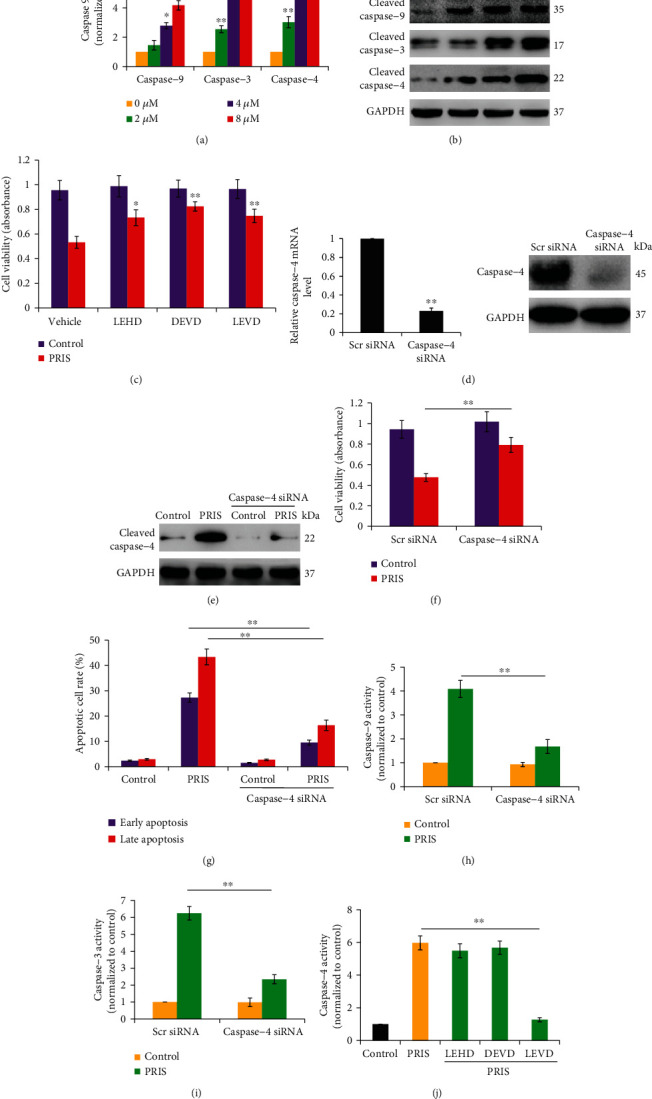
PRIS induces caspase-dependent intrinsic apoptotic cell death in CRLCs. (a) CRLCs were treated with 0 (control), 2, 4, and 8 *μ*M PRIS for 12 h; caspase assay was performed to measure the activity of caspase-9, caspase-3, and caspase-4 (*n* = 5). (b) Cells were treated as described above in (a); relative changes of protein levels in cytochrome c, cleaved caspase-9, cleaved caspase-3, and cleaved caspase-4 were analyzed by Western blot. (c) Cells were incubated with caspase inhibitors (10 *μ*M Z-LEHD-FMK for caspase-9, 10 *μ*M Z-DEVD-FMK for caspase-3, and 10 *μ*M Ac-LEVD-CHO for caspase-4) for 1 h, followed by treatment with PRIS (4 *μ*M) for 24 h. Cell viability was measured by MTS assay (*n* = 6). (d) CRLCs were transfected with caspase-4-specific or nonspecific siRNA for 48 h, and mRNA and protein expression was measured to determine the efficiency of the silence. (e, f) CRLCs were incubated in the absence or presence of caspase-4 siRNA for 48 h. Then, cells were treated by PRIS (4 *μ*M) (or not) and cell viability or protein expression was determined by MTS assay (*n* = 5) or Western blot. (g) CRLCs were incubated in the absence or presence of caspase-4 siRNA for 48 h. Then, cells were treated by PRIS (8 *μ*M) and cell apoptosis was assessed by flow cytometry using Annexin V/PI double staining. (h, i) Cells were treated with scramble siRNA or siRNA to caspase-4 for 48 h and then exposed to PRIS (8 *μ*M) for 24 h. Caspase activity was determined using specific substrates (*n* = 6). (j) CRLCs were preincubated with caspase inhibitors (10 *μ*M Z-LEHD-FMK for caspase-9, 10 *μ*M Z-DEVD-FMK for caspase-3, and 10 *μ*M Ac-LEVD-CHO for caspase-4) for 1 h, followed by treatment with PRIS (8 *μ*M) for 24 h. The activities of caspase-4 were monitored via caspase assay (*n* = 5). All Western blot band intensities were normalized to GAPDH. Data are presented as mean ± SD, ∗*P* < 0.05, ∗∗*P* < 0.01.

Our previous report demonstrated that PRIS induced significant cell apoptosis in a dose-dependent manner in CRLCs [34]. Because caspases are critical molecules of apoptotic cell death which are activated by cytochrome c, we investigated the effect of PRIS on release of cytochrome c and caspase activation (cleavage). Exposure of 2, 4, and 8 *μ*M of PRIS significantly increased the activities of caspase-9, caspase-3, and caspase-4 in a dose-dependent manner by the caspase activity assay, and an increase in the release of cytochrome c and cleaved fragments of caspase-9, caspase-3, and caspase-4 was observed upon PRIS treatment for 12 h (Figures 4(a) and 4(b)). To investigate whether caspase activation affected PRIS-induced apoptotic cell death, cells were preincubated with caspase inhibitors (Z-LEHD-FMK for caspase-9, Z-DEVD-FMK for caspase-3, and Ac-LEVD-CHO for caspase-4) for 1 h, followed by treatment with PRIS (4 *μ*M) for 24 h. All of caspase inhibitors significantly inhibited PRIS-induced cell death in CRLCs (Figure 4(c)). To confirm whether caspase-4 is a critical factor in PRIS-induced apoptosis in CRLCs, cells were transfected with caspase-4 siRNA for 48 h, followed by treatment with PRIS for 24 h. RT-PCR and Western blot analysis were performed to ensure adequate knocking down of capase-4 (Figure 4(d)). Compared with those of scrambled siRNA-transfected cells, knockdown of caspase-4 significantly attenuated PRIS-induced cleaved caspase-4 expression and cell death (Figures 4(e) and 4(f)). Moreover, caspase-4 knockdown markedly decreased PRIS-triggered early and late apoptosis in CRLCs (Figure 4(g)). Furthermore, our results revealed that siRNA against caspase-4 suppressed PRIS-induced activation of caspase-9 and caspase-3 (Figures 4(h) and 4(i)). However, PRIS-induced enhancement of caspase-4 activity was not attenuated by Z-LEHD-FMK (caspase-9 inhibitor) or Z-DEVD-FMK (caspase-3 inhibitor) (Figure 4(j)). Taken together, these results indicate that caspase-4 plays an important role in PRIS-induced cell death through activating the intrinsic (mitochondrial-mediated) apoptotic pathway.

### 3.5. PRIS Induces ER Stress in CRLCs

**Figure 5:**
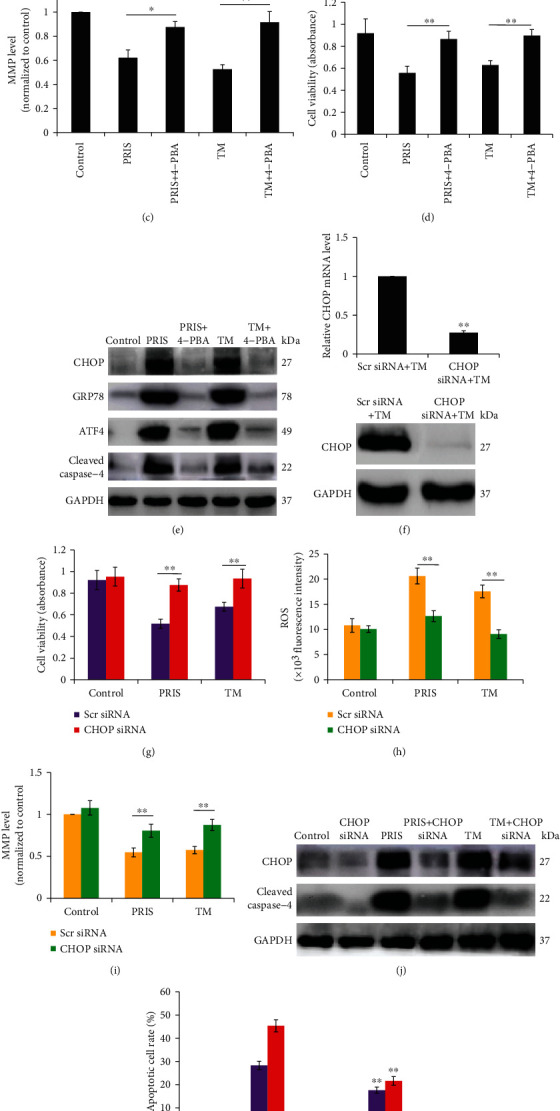
PRIS induces ER stress in CRLCs. (a, b) CRLCs were treated with 0 (control), 2, 4, and 8 *μ*M PRIS or 2 *μ*M Tunicamycin (TM) for 24 h; cell lysates were resolved by SDS-PAGE and analyzed by Western blot with antibodies against CHOP, GRP78, ATF4, and GAPDH. (b) CRLCs were treated with 0 (control), 2, 4, and 8 *μ*M PRIS or 2 *μ*M TM for 6 h; cell lysates were resolved by SDS-PAGE and analyzed by Western blot with antibodies against p-eIF2*α*, eIF2*α*, p-IRE1*α*, and IRE1*α*. (c–e) Cells were pretreated with 4-PBA (1 mM) for 90 min and followed by treatment with 4 *μ*M PRIS or 2 *μ*M TM for 24 h. After each treatment, cells were incubated with 20 nM DiOC_6_ for 30 min and MMP was measured by flow cytometry (*n* = 5). Cellular viability was determined by MTS assay (*n* = 5). Cell lysates were analyzed by Western blot with antibodies against CHOP, GRP78, ATF4, cleaved caspase-4, and GAPDH. (f) CRLCs were transfected with CHOP-specific or nonspecific siRNA. 48 h after transfection, mRNA and protein expression was measured to determine the efficiency of the silence. (g) CRLCs were incubated in the absence or presence of CHOP siRNA for 48 h, then treated with PRIS (4 *μ*M) or TM (2 *μ*M), and cellular viability was determined by MTS assay (*n* = 7). (h–j) Cells were treated as described above in (g); production of ROS was quantified by the amount of cellular DCF synthesis. The fluorescence intensity was quantified using a fluorescence microplate reader (*n* = 6). MMP level was measured by flow cytometry (*n* = 5), and indicated protein expressions were detected by Western blot. (k) CRLCs were transfected with CHOP-specific or nonspecific siRNA, then incubated with PRIS (8 *μ*M) for 24 h. Vehicle- or PRIS-treated cells were stained with Annexin V-FITC and PI and evaluated by flow cytometry (*n* = 4). All Western blot band intensities were normalized to the total proteins or GAPDH. Data are presented as mean ± SD, ∗*P* < 0.05, ∗∗*P* < 0.01.

Because caspase-4 is shown to be closely related to ER stress-induced cell death, we postulated that PRIS may cause ER stress. To determine whether PRIS can increase the expression and activation of ER stress-associated proteins, CRLCs were treated with PRIS or Tunicamycin (TM) as a positive control for ER stress induction [35]. Western blot analysis was performed to examine the effects of PRIS on the expression of ER stress-associated proteins, such as CHOP, GRP78, and ATF4. Our results showed increased expression of CHOP and GRP78, as well as stimulated ATF4 at 24 h in cells treated with PRIS (Figure 5(a)). In addition, the effects of PRIS on the ER stress-derived initial unfolded protein response were examined, including the phosphorylation of eIF2*α* and IRE1*α*. As shown in Figure 5(b), PRIS induced an increase in the phosphorylation of eIF2*α* and IRE1*α* at 6 h. To understand whether ER stress is involved in PRIS-induced apoptosis and how they interact with each other, cells were preincubated with 4-phenylbutyric acid (4-PBA), which acts as an authentic chemical chaperone by aiding protein folding in the ER [36], followed by treatment with PRIS or TM for 24 h. The results showed that 4-PBA recovered the MMP levels decreased by PRIS when compared with PRIS or TM-treated groups (Figure 5(c)). Moreover, pretreatment with 4-PBA significantly reversed PRIS-induced cell death and CHOP expression as well as caspase-4 cleavage in CRLCs (Figures 5(d) and 5(e)). These results suggest that ER stress may contribute to PRIS-induced mitochondrial dysfunction and subsequent cell death. To confirm the role of CHOP in PRIS-induced apoptotic cell death, CHOP expression was blocked by transfection with CHOP siRNA for 48 h, and the effects of PRIS on cell viability were then examined at 24 h. RT-PCR and Western blot analysis were performed to ensure adequate silencing (Figure 5(f)). CHOP knockdown resulted in a significant reduction of PRIS- or TM-induced cell death and ROS production, as well as increased MMP level (Figures 5(g)–5(i)). Furthermore, downregulation of CHOP dramatically decreased the caspase-4 cleavage and caused a decreased apoptosis rate in PRIS-treated CRLCs (Figures 5(j) and 5(k)). These data suggest that PRIS-induced CHOP expression might be responsible for apoptotic cell death, and ER stress plays a key role in PRIS-induced cell death in human CRLCs.

### 3.6. PRIS Induces Mitochondrial Dysfunction via ER Stress Responses in CRLCs

**Figure 6:**
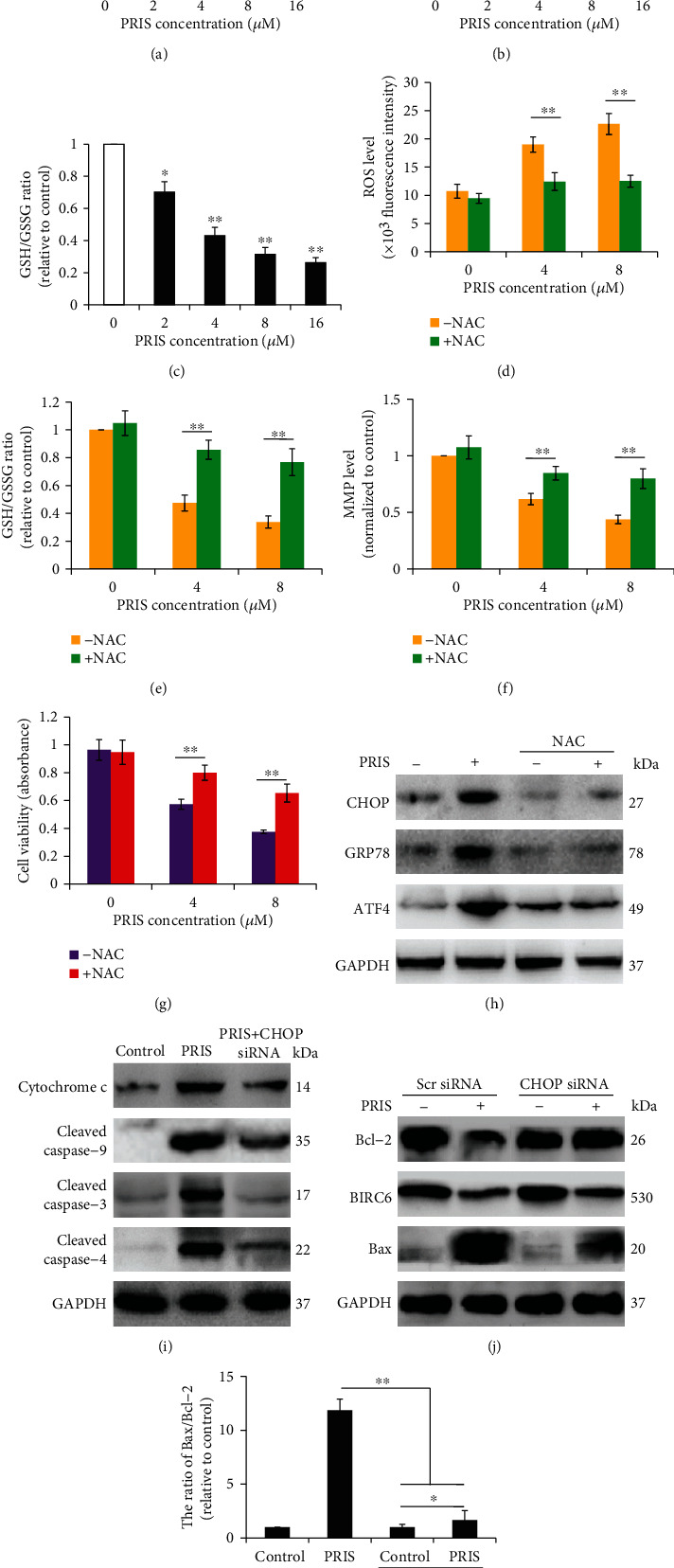
PRIS induces mitochondrial dysfunction via ER stress responses in CRLCs. Cells were treated with 2, 4, 8, and 16 *μ*M PRIS for 24 h. (a) ROS generation was determined by the amount of cellular DCF formation (*n* = 6). (b) After treatment, cells were incubated with 20 nM DiOC_6_ for 30 min and MMP was measured by flow cytometry (*n* = 6). (d, e) CRLCs were pretreated with 5 mM NAC for 60 min, then incubated with 4 or 8 *μ*M PRIS for 24 h; ROS generation was determined by the amount of cellular DCF formation (*n* = 5), and GSH/GSSG was measured and the ratio was calculated (*n* = 6). (f, g) Cells were treated as described above in (d, e), then assessed for MMP by flow cytometry (*n* = 6). Cell viability was determined by MTS assay (*n* = 5). (h) CRLCs were pretreated with 5 mM NAC for 60 min, then incubated with 4 *μ*M PRIS for 24 h, and indicated protein expressions were measured by Western blot. (i–k) Cells were transfected with scrambled (Scr) siRNA or CHOP siRNA for 48 h and then treated with 4 *μ*M PRIS for 24 h, and indicated protein expression was detected by Western blot. All Western blot band intensities were normalized to GAPDH. Data are presented as mean ± SD, ∗*P* < 0.05, ∗∗*P* < 0.01.

Accumulating evidence has indicated that mitochondrial dysfunction is quite important in modulating responses to cancer therapeutic agents. Mitochondrial dysfunctions are responsible for ER stress-associated apoptotic cell death [37]. We therefore determined whether ROS generation is involved in the growth inhibition and proapoptotic effect elicited by PRIS. As shown in Figures 6(a) and 6(b), incubation of CRLCs with PRIS markedly increased intracellular ROS levels as indicated by the increased levels of DCF fluorescence, while MMP declined remarkably. In addition, GSH and GSSG levels were quantified to confirm the effect of PRIS on ROS generation. Because GSH is reversibly oxidized to GSSG under oxidative conditions, reduction of the GSH/GSSG ratio is used to measure ongoing oxidative stress [38]. The GSH to GSSG ratio was significantly decreased in CRLCs after PRIS treatment (Figure 6(c)). These data indicated that PRIS could affect ROS generation and alter the redox status of CRLCs. To further determine whether ROS participates in PRIS-induced ER stress and its relevant biofunctions, cells were pretreated with N-Acetyl-L-cysteine (NAC) prior to PRIS incubation. We found that pretreatment of the CRLCs with NAC at a concentration of 5 mM abrogated PRIS-induced ROS generation and increased the GSH/GSSG ratio, respectively (Figures 6(d) and 6(e)). NAC also significantly blocked PRIS-induced MMP loss and attenuated PRIS-induced cytotoxicity (Figures 6(f) and 6(g)). Furthermore, the NAC pretreatment significantly decreased CHOP, GRP78, and ATF4 expression induced by PRIS (Figure 6(h)). As shown in Figures 6(i)–6(k), downregulation of CHOP strongly suppressed the PRIS-stimulated cytochrome c release and inhibited the cleavage of caspase-9, caspase-3, and caspase-4 and the ratio expression of Bax/Bcl-2. Furthermore, the expression of BIRC6 was more markedly increased when CHOP was silenced. These results suggested that ER stress may contribute to PRIS-induced mitochondrial dysfunction and subsequent cell death as downstream of ROS.

### 3.7. The EphB4/CDC42/N-WASP Pathway Plays a Role in PRIS-Induced Cell Death in CRLCs

**Figure 7:**
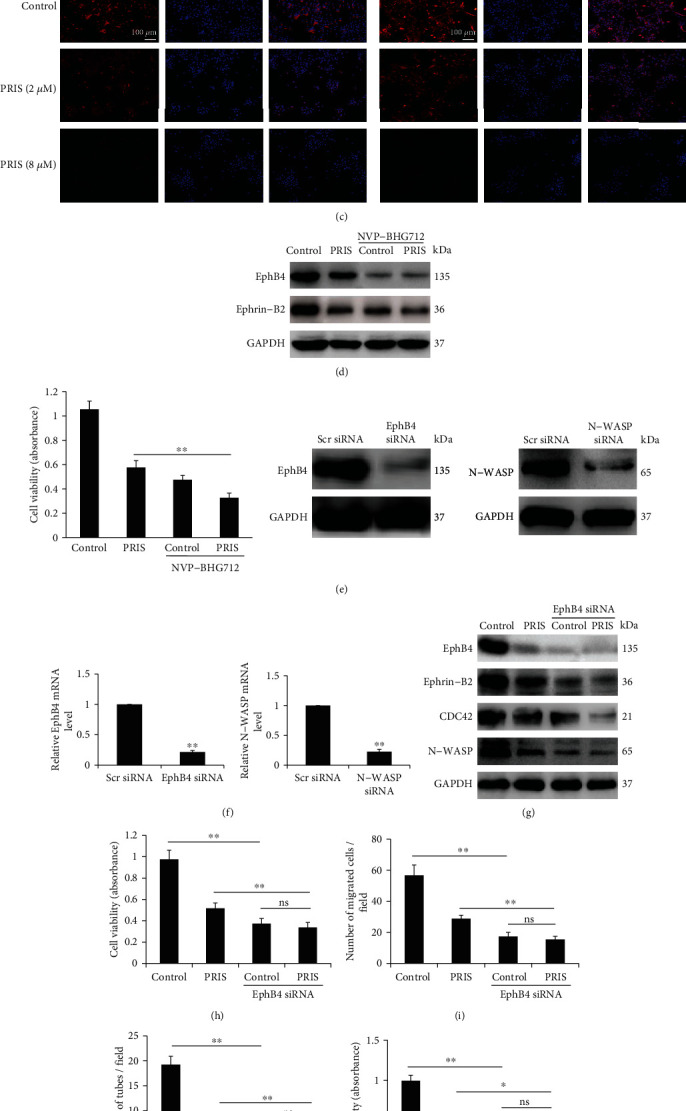
EphB4/CDC42/N-WASP signaling plays a role in PRIS-induced cell death in CRLCs. (a) Protein expression of EphB4 was detected by Western blot in BEAS-2B cells and CRLCs derived from the tumor tissue of different patients. (b) CRLCs were treated with 0 (control), 2, 4, and 8 *μ*M PRIS for 24 h; cell lysates were resolved by SDS-PAGE and analyzed by Western blot with antibodies against EphB4, Ephrin-B2, CDC42, N-WASP, and GAPDH. (c) Cells were treated as described in (b); EphB4 and N-WASP were stained with primary antibodies followed by staining with secondary DyLight® 594-conjugated antibodies. Cell nuclei were labeled with DAPI, and immunofluorescence analysis was performed under the fluorescence microscope. (d, e) PRIS (4 *μ*M) alone or combined with NVP-BHG712 (0.1 *μ*M) was applied to CRLCs, and Western blot analysis and cellular viability assay were performed. (f) CRLCs were transfected with EphB4-specific and N-WASP-specific or nonspecific siRNA for 48 h. mRNA and protein expression was measured to determine the efficiency of the silence. (g) CRLCs were incubated in the absence or presence of EphB4 siRNA for 48 h. Then, cells were treated by PRIS (4 *μ*M) and indicated protein expressions were measured by Western blot. (h, k) Cells were transfected with scrambled (Scr) control, EphB4, or N-WASP siRNA for 48 h and then treated with 4 *μ*M PRIS for 24 h; cell viability was determined by MTS assay (*n* = 6). (i, l) CRLCs were treated under the same conditions as described above in (h, k), and cell migration and invasion ability were determined by Transwell assay. The numbers of migrated cells were quantified by performing cell counts of 10 random fields (*n* = 4). (j, m) CRLCs were treated under the same conditions as described above in (h, k); HUVECs treated by conditioned medium were seeded on a Matrigel-precoated 96-well plate, and capillary-like tube formation was observed under an inverted light microscope (*n* = 5). All Western blot band intensities were normalized to GAPDH. Data are presented as mean ± SD, ∗*P* < 0.05, ∗∗*P* < 0.01. ns: not significant.

EphB4 expression is elevated in a variety of human cancers, including cancers of the head and neck, ovaries, lung, and esophagus [39–42]. We analyzed the expression of EphB4 in a panel of CRLCs from different patient derived-samples compared to its expression in BEAS-2B, which was isolated from normal human bronchial epithelium. Both mRNA and protein analysis revealed that the EphB4 is overexpressed in all CRLCs from tumor samples tested compared with normal human bronchial epithelial cells (Figure 7(a)). To determine the effect of PRIS on the EphB4 signaling pathway, Western blot and immunofluorescence staining were used to examine the protein expression from CRLCs treated with various concentrations (0, 2, 4, and 8 *μ*M) of PRIS for 24 h. As shown in Figures 7(b) and 7(c), PRIS markedly inhibited the expression of EphB4 and Ephrin-B2. Moreover, the expression of CDC42 and N-WASP was significantly decreased after PRIS (2 and 8 *μ*M) pretreatment for 24 h in CRLCs. Previous studies have investigated the role of EphB4 in lung cancer and reported that EphB4 is expressed more strongly in tumor tissues compared to paired normal samples, and knockdown or inhibition of EphB4 attenuates the growth of cancer cells *in vitro* and *in vivo* [41, 43]. We thus hypothesized that EphB4/Ephrin-B2 inhibition is responsible for the decreased cell survival observed in response to PRIS and performed inhibiting experiments by use of NVP-BHG712, a specific kinase inhibitor of the EphB4. As shown in Figure 7(d), NVP-BHG712 (0.1 *μ*M) remarkably suppressed EphB4 and Ephrin-B2 expression and abolished the prevention by PRIS on them. Furthermore, the presence of NVP-BHG712 significantly suppressed the cell viability and enhanced the cytotoxic effect imposed by PRIS (Figure 7(e)). To determine the role of EphB4 and N-WASP in the context of PRIS-mediated cytotoxic effects, we used specific siRNA to silence the EphB4 and N-WASP expression in CRLCs. RT-PCR and Western blot analysis were performed to ensure adequate silencing (Figure 7(f)). As shown in Figure 7(g), the expression of Ephrin-B2, CDC42, and N-WASP was largely inhibited when cells were silenced with EphB4 compared with cells transfected with the nonspecific siRNA. Notably, cell viability was significantly decreased while the inhibition of cell migration and capillary-like structure formation were considerably augmented in case of EphB4 knockdown (Figures 7(h)–7(j)). Furthermore, knockdown of N-WASP suppressed cell survival and inhibited the migrative and tube formation ability of CRLCs, illustrating that loss of N-WASP is cytotoxic during PRIS exposure (Figures 7(k)–7(m)). More importantly, the antitumor effect of PRIS was significantly abolished in case of EphB4 or N-WASP knockdown. All together, these results demonstrated that PRIS exerts its cytotoxic potential via the EphB4/CDC42/N-WASP signaling pathway.

### 3.8. PRIS Induces ER Stress-Mediated Intrinsic Cell Apoptosis via EphB4/CDC42/N-WASP Signaling

**Figure 8:**
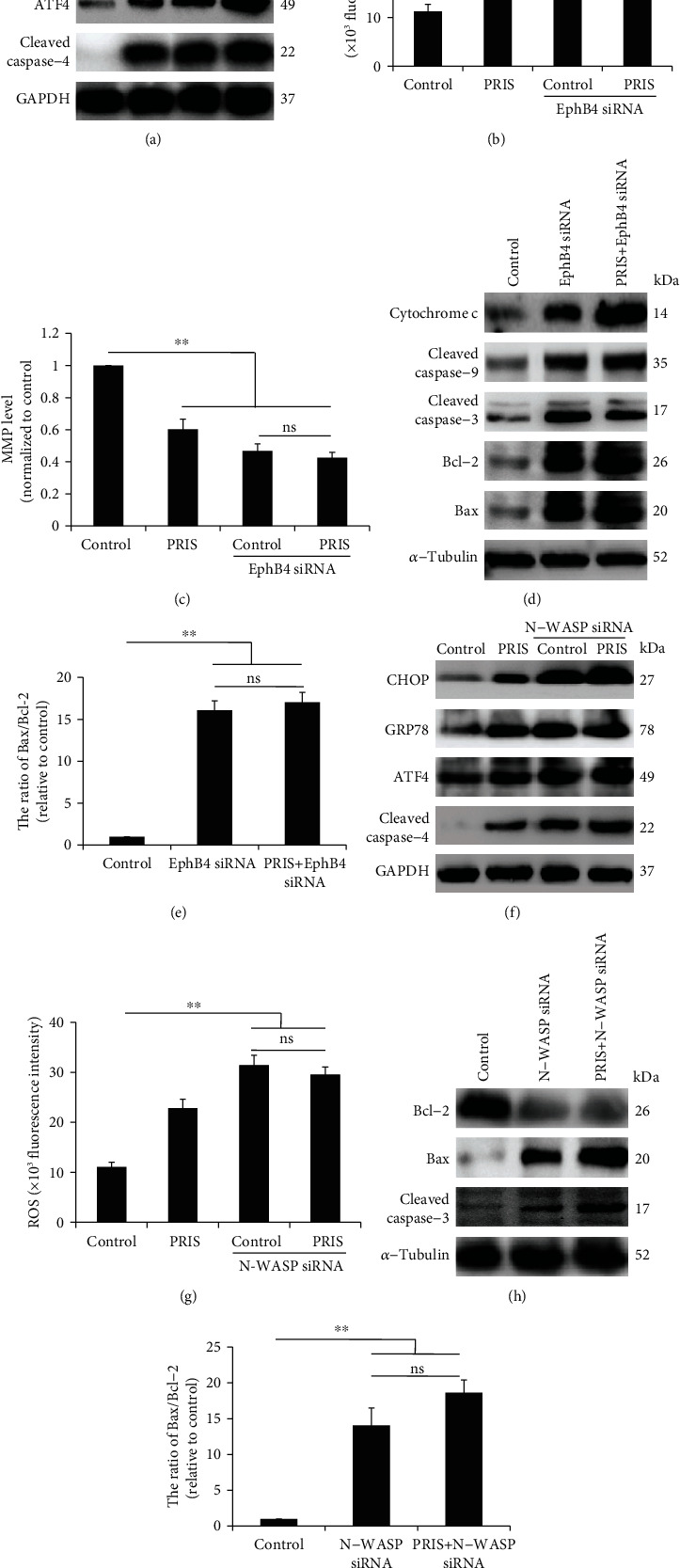
PRIS induces ER stress-mediated intrinsic cell apoptosis via EphB4/CDC42/N-WASP signaling. (a) CRLCs were incubated in the absence or presence of EphB4 siRNA for 48 h. Then, cells were treated by 4 *μ*M PRIS and indicated protein expressions were measured by Western blot. (b, c) CRLCs were treated under the same conditions as described above in (a); ROS generation was determined by the amount of cellular DCF formation (*n* = 6), and MMP level was detected by flow cytometry (*n* = 6). (d, e) Cells were transfected with scrambled (Scr) siRNA or EphB4 siRNAs for 48 h and then treated with 4 *μ*M PRIS for 24 h. Relative changes in cytochrome c, cleaved caspase-9 and caspase-3, Bcl-2, and Bax protein levels were analyzed by Western blot. (f) CRLCs were incubated in the absence or presence of N-WASP siRNA for 48 h. Then, cells were treated by 4 *μ*M PRIS and indicated protein expressions were measured by Western blot. (g) Cells were treated as described above in (f); production of ROS was quantified by the amount of cellular DCF synthesis. The fluorescence intensity was quantified using a fluorescence microplate reader (*n* = 5). (h, i) Cells were transfected with scrambled (Scr) siRNA or N-WASP siRNA for 48 h and then treated with 4 *μ*M PRIS for 24 h. Relative changes in Bcl-2, Bax, and cleaved caspase-3 protein levels were analyzed by Western blot. All Western blot band intensities were normalized to GAPDH or *α*-tubulin. Data are presented as mean ± SD, ∗*P* < 0.05, ∗∗*P* < 0.01. ns: not significant.

Previous report revealed that EphB4 inhibition induced prostate cancer cell death via activation of ER stress [44]. Based on the findings discussed above, we hypothesized that the EphB4/CDC42/N-WASP signaling may be involved in the promotion of ER stress exerted by PRIS. Therefore, we detected the effect of PRIS on ER stress-associated proteins in case of EphB4 knockdown. As shown in Figure 8(a), inhibition of EphB4 by siRNA transfection significantly induced ER stress-associated CHOP, GRP78, and ATF4 expression, as well as caspase-4 cleavage. In addition, knockdown of EphB4 increased ROS production (152.1% of nonsilenced PRIS group, *P* < 0.01) and repressed MMP level, implying that loss of EphB4 may induce mitochondrial dysfunction in CRLCs (Figures 8(b) and 8(c)). To further confirm the results derived from the cytotoxicity assay, we examined the protein levels of CRLCs and found that the inhibition of EphB4 significantly increased the release of cytochrome c, enhanced the induction of cleaved caspase-9 and caspase-3 protein levels, and elevated the Bax/Bcl-2 ratio expression (Figures 8(d) and 8(e)). Strikingly, inhibition of EphB4 by siRNA transfection completely abolished the induction by PRIS on cleaved caspase-3 and Bax/Bcl-2 expression (Figure 8(d)). In an effort to confirm the possible role of N-WASP in the promotion of ER stress, we showed that siRNA transfection of N-WASP upregulated the expression of CHOP, GRP78, and ATF4, promoted cleaved caspase-4 and caspase-3 activation, elevated the Bax/Bcl-2 ratio expression, and increased significant ROS production (Figures 8(f)–8(i)).

In summary, the combined data supported that activation of the EphB4/CDC42/N-WASP signaling pathway plays a key role in PRIS-induced cell death that is mediated by ER stress.

## 4. Discussion

Despite progress in locoregional and systemic therapies, patient survival from lung cancer remains a challenge. Given the inefficacy in the current therapeutic strategies for lung cancer, the characterization of the key aberrantly deregulated signal pathways in initiating and maintaining the lung cancer development and progression is a critical step in innovating even revolutionizing our current lung cancer therapeutic choices. New alternative therapeutic agents targeting proliferation of cancer cells are attracting attention in recent years. In the present study, we established and characterized patient-derived CRLCs to assess their biological properties and to apply these to test the efficacies of PRIS. These new primary culture technologies consist of CR cells cocultured as monolayers with feeder cells (irradiated-3T3 mouse fibroblasts) in the presence of a Rho-associated protein kinase inhibitor (Y27632) that provides a unique platform for identifying new approaches for early therapeutic intervention. The possible mechanism that is operative in the generation of these indefinite proliferative CRCs with combination of F medium containing feeder cells and Y-27632 would be induction of telomerase and cytoskeletal remodeling and/or interference with the p16/Rb pathway [26]. This reprogramming of cell growth and differentiation is conditional which are independent of exogenous viral or cellular gene transduction, thereby maintaining critical biological and genetic characteristics as compared with those of the primary tissues than cells immortalized by human telomerase reverse transcriptase [27]. In tumor CR cell cultures in our current study, phenotypic and genotypic features of the primary tumor are maintained, which is corresponding to the previous report [26]. More importantly, CR conditions allow immortalization of our cultured primary lung cancer cells while preserving their tumorigenic potential.

In the present study, we found that PRIS inhibited the growth of CRLCs by suppressing key functional activities including cellular proliferation, migration, invasion, and capillary structure formation. Moreover, PRIS induced caspase-4-dependent intrinsic apoptosis in CRLCs via ER stress and mitochondrial dysfunction through the EphB4/CDC42/N-WASP signaling pathway.

In addition to suppression of cell growth and survival activities, stimulation of proapoptotic and apoptotic cascades is probably essential to ensure cells entering into programmed cell death. PRIS was reported to induce apoptosis through mitochondrial dysfunction in several types of cancer cells such as HepG2 hepatocellular carcinoma cells, cervical cancer cells, and glioma cells [45–47]. A reduction in MMP may trigger apoptosis, and it is usually considered as an earlier event before cytochrome c and other mitochondrial factors in the apoptosis cascade. In the present study, we found that PRIS reduced MMP in human CRLCs, suggesting that mitochondrial dysfunction may be a generalized mechanism in PRIS-induced apoptosis. Apoptosis as a regulated mode of cell death includes two major pathways, the death-receptor-mediated extrinsic pathway and the mitochondria-dependent intrinsic pathway [48]. Mitochondria have been shown to play a central role in the apoptotic process, through both intrinsic and extrinsic pathways [49]. Mitochondria-mediated apoptosis is characterized by the collapse of the MMP, resulting in the release of mitochondrial proteins such as cytochrome c, thus triggering the activation of caspases typically represented by caspase-9, caspase-7, and caspase-3. This essential process is characteristically activated by proapoptotic Bax and inhibited by antiapoptotic Bcl-2 and Bcl-xL. Consistent with the reports which provided evidence that the mitochondrial depolarization in lung cancer cells was due to the imbalance of Bax/Bcl-2 [50], we found that treatment with PRIS increased the cellular level of proapoptotic Bax and reduced the levels of antiapoptotic Bcl-2. This led to the decline of MMP and activation of cytochrome c release of CRLCs, which in turn exacerbated the mitochondrion-mediated apoptosis. Moreover, PRIS also decreased expression of BIRC6, which is a caspase inhibitor that has recently been shown to be overexpressed in different types of cancer such as lung cancer [51]. Furthermore, we found that PRIS triggered the activation of caspase-9 and caspase-3, which are modulators of the mitochondrial pathway (intrinsic).

Procaspase-9 can be processed by active caspase-4, which is a member of caspase-1 subfamily that is localized to the ER membrane and is cleaved when cells are treated with ER stress-inducing reagents, but not with other apoptotic reagents [52]. Caspase-4 can function as an ER stress-specific caspase in humans and is primarily activated in ER stress-mediated apoptosis in various types of cells such as human carcinoma HeLa cells and human neuroblastoma SK-N-SH cells [52, 53]. In addition, caspase-4 acts upstream of caspase-9 in TM-induced apoptosis in human neuroblastoma SH-SY5Y cells [53]. The present study showed that in addition to caspase-9 and caspase-3, PRIS also induced cleavage and activation of caspase-4 in CRLCs. Caspase-4 knockdown by siRNA blocked PRIS-induced cell death. Therefore, we assessed the role of caspase-4 in PRIS-induced caspase activation and found that inhibition of caspase-4 by siRNA was associated with reduced activation of caspase-9 and caspase-3. However, inhibition of caspase-9 and caspase-3 using pharmacological inhibitors did not lead to changes in caspase-4 activity. These findings suggest that caspase-4 activation is an important step in PRIS-induced activation of caspase-9 and caspase-3.

ER stress is known to be a critical initiator and activator of cell death in pathological conditions, which is implicated in the pathogenesis of a variety of diseases (neurodegeneration, inflammation, and cancer) [54, 55]. Emerging evidence indicates that pharmacological targeting of ER stress can represent an effective therapeutic strategy to treat tumors [56]. Under misfolded protein stress, the ER activates the unfolded protein response (UPR) to achieve remission of the stress and regain homeostasis of ER. However, severe and sustained ER stress would further contribute to apoptosis by inducing activation of proapoptotic factor CHOP which would activate caspase-4 and lead to caspase cascade activation [37, 57, 58]. In this regard, we showed that PRIS triggered ER stress by significantly enhancing the protein levels of various ER stress-associated proteins, such as CHOP, GRP78, ATF4, p-eIF2*α*, and p-IRE1*α*, with levels similar to TM induction. Previous study has demonstrated that PRIS activates ASK1/JNK signaling induced by ROS promotion which results in cell apoptosis and autophagy in human breast cancer [11]. Accumulating evidence suggests the interrelation of ER stress and ROS with redox signaling mediators such as protein disulfide isomerase- (PDI-) endoplasmic reticulum oxidoreductin- (ERO-) 1, GSH/GSSG, NADPH oxidase 4 (Nox4), and calcium [59]. Generally, ROS- and ER stress-mediated apoptotic events funnel through mitochondria [60]. ROS plays an important role in the process of cancer genesis and metastasis, and accumulation of ROS due to chemotherapeutic agents is known to cause persistent ER stress and mitochondrial dysfunction. In our study, ROS inhibitor NAC blocked the PRIS-induced activation of ER stress proteins, such as CHOP, GRP78, and ATF-4, which revealed that ROS generation is an early trigger of PRIS-activated ER stress. Conversely, the very close proximity of ER and mitochondria leads to accumulation of Ca^2+^ near mitochondria, thereby increasing mitochondrial ROS production [59, 61]. Because both mitochondrial and ER events are implicated in PRIS-induced cell death, we examined how ER stress and mitochondrial dysfunction crosstalk each other in PRIS-induced death pathways. The results demonstrated that inhibition of ER stress by pharmacological and molecular means prevented PRIS-induced ROS production and MMP decline and reduced PRIS-induced apoptotic cell death, which suggests that ER stress concurrently plays a role in mitochondrial dysfunction in PRIS-treated CRLCs. These results provide novel evidence of positive feedback regulation mechanisms between PRIS-induced ROS and mitochondrial dysfunction or ER stress pathway.

In the lung, microarray data from previous studies indicated that EphB4 is upregulated with lung cancer progression when comparing normal versus adenocarcinoma samples [62]. Existing evidence also showed that EphB4 was expressed on the surface of breast cancer cells that promote angiogenesis in tumor xenografts by activating Ephrin-B2 reverse signaling in the vasculature, thus increasing tumor growth [63]. EphB4 siRNA significantly inhibited tumor cell viability, induced apoptosis, activated caspase-8, and inhibited the growth of tumor xenografts *in vivo* in head and neck squamous cell carcinoma [64]. Inhibition of EphB4 promoted tumor cell growth which was mediated by the activation of ER stress in prostate cancer [44]. In the present study, we analyzed both mRNA and protein expression of EphB4 in CRLCs from clinical specimens of lung adenocarcinoma, and all samples revealed persistent EphB4 expression. Interestingly, we found that inhibition of EphB4 by siRNA transfection enhanced PRIS-induced caspase-4 cleavage/activation, ER stress-associated protein expression, and subsequent cell death in CRLCs. Consistent with its effects on cell viability, knockdown of EphB4 further enhanced PRIS-induced ROS production and MMP loss in CRLCs, suggesting that EphB4 is involved in PRIS-induced ER stress and mitochondrial dysfunction. In addition, knockdown of EphB4 decreased expression of proapoptotic Bcl-2 family members and enhanced activation of caspase-9, an initiator caspase of the mitochondrial/intrinsic pathway. Similar to its role in the embryonic vasculature, Ephrin-B2 signaling may promote remodeling of immature tumor vascular networks, with the pruning of some vessels and enlargement of others [65]. Thus, we propose a role for Ephrin-B2 in promoting efficient tumor vascularization. We showed in the current study that PRIS markedly inhibited Ephrin-B2 expression, and silencing of EphB4 resulted in impaired activation of Ephrin-B2 and reduced cell migration and capillary tube formation. Previous study has demonstrated the ability of Eph receptors to activate different Rho-GTPases, such as RhoA and CDC42 [63]. CDC42 is crucial for EGF- stimulated migration in MTLn3 carcinoma cells [66]. And CDC42 and N-WASP are critical for the formation of invadopodia, which combines localized actin protrusion with matrix metalloproteinase (MMP) secretion to degrade extracellular matrices and allow invasion [67]. In the current study, PRIS treatment was found to decrease the expression of CDC42 and N-WASP. The knockdown of N-WASP expression led to an increase in ROS generation and cell death, as well as an elevated level of ER stress and Bax/Bcl-2 ratio expression. These findings highlight the crucial role of the EphB4-Ephrin-B2/CDC42/N-WASP signaling in the suppression of cell growth, migration, and tube formation in CRLCs mediated by PRIS.

## 5. Conclusions

In conclusion, we established and characterized CRLCs in the present study to assess their biological properties and to apply these to test the efficacies of PRIS. We firstly demonstrated in this study that PRIS suppressed CRLCs growth by inhibiting cell viability, migration, invasion, and capillary structure formation. From a mechanistic standpoint, the downregulation of EphB4/CDC42/N-WASP signaling was potentially responsible for mitochondrial-mediated intrinsic apoptosis and activation of ER stress induced by PRIS, consequently resulting in cellular injury in CRLCs. These data shed light on molecular processes underlying the oxidative and ER stress signaling cascade engaged by PRIS and identify potential targets for intervention to prevent lung adenocarcinoma.

## Data Availability

The data used to support the findings of this study are available from the corresponding author upon request.

## Supplementary Materials

Supplementary Materials This section includes (1) original data.docx, (2) ROS-si-EphB4-original.xlsx, and (3) ROS-si-N-WASP-original.xlsx.

## References

[1] Siegel R. L., Miller K. D., Jemal A. (2016). Cancer statistics, 2016. *CA: a Cancer Journal for Clinicians*.

[2] Yousef B. A., Hassan H. M., Guerram M. (2016). Pristimerin inhibits proliferation, migration and invasion, and induces apoptosis in HCT-116 colorectal cancer cells. *Biomedicine & pharmacotherapy = Biomedecine & pharmacotherapie*.

[3] Brinker A. M., Ma J., Lipsky P. E., Raskin I. (2007). Medicinal chemistry and pharmacology of genus Tripterygium (Celastraceae). *Phytochemistry*.

[4] Yousef B., Hassan H., Zhang L.-Y., Jiang Z.-Z. (2017). Anticancer potential and molecular targets of pristimerin: a mini-review. *Current Cancer Drug Targets*.

[5] Yousef B. A., Guerram M., Hassan H. M., Hamdi A. M., Zhang L. Y., Jiang Z. Z. (2016). Pristimerin demonstrates anticancer potential in colorectal cancer cells by inducing G1 phase arrest and apoptosis and suppressing various pro-survival signaling proteins. *Oncology Reports*.

[6] Huang S., He P., Peng X., Li J., Xu D., Tang Y. (2015). Pristimerin inhibits prostate cancer bone metastasis by targeting PC-3 stem cell characteristics and VEGF-induced vasculogenesis of BM-EPCs. *Cellular Physiology and Biochemistry: International Journal of Experimental Cellular Physiology, Biochemistry, and Pharmacology*.

[7] Deeb D., Gao X., Liu Y. B., Pindolia K., Gautam S. C. (2014). Pristimerin, a quinonemethide triterpenoid, induces apoptosis in pancreatic cancer cells through the inhibition of pro-survival Akt/NF-*κ*B/mTOR signaling proteins and anti-apoptotic Bcl-2. *International Journal of Oncology*.

[8] Byun J. Y., Kim M. J., Eum D. Y. (2009). Reactive oxygen species-dependent activation of Bax and poly(ADP-ribose) polymerase-1 is required for mitochondrial cell death induced by triterpenoid pristimerin in human cervical cancer cells. *Molecular Pharmacology*.

[9] Lu Z., Jin Y., Chen C., Li J., Cao Q., Pan J. (2010). Pristimerin induces apoptosis in imatinib-resistant chronic myelogenous leukemia cells harboring T315I mutation by blocking NF-*κ*B signaling and depleting Bcr-Abl. *Molecular Cancer*.

[10] Cevatemre B., Erkısa M., Aztopal N. (2018). A promising natural product, pristimerin, results in cytotoxicity against breast cancer stem cells in vitro and xenografts in vivo through apoptosis and an incomplete autopaghy in breast cancer. *Pharmacological Research*.

[11] Zhao Q., Liu Y., Zhong J. (2019). Pristimerin induces apoptosis and autophagy via activation of ROS/ASK1/JNK pathway in human breast cancer in vitro and in vivo. *Cell Death Discov*.

[12] Banerjee A., Lang J. Y., Hung M. C. (2011). Unfolded protein response is required in nu/nu mice microvasculature for treating breast tumor with tunicamycin. *The Journal of Biological Chemistry*.

[13] Zhu X., Huang L., Gong J. (2017). NF-*κ*B pathway link with ER stress-induced autophagy and apoptosis in cervical tumor cells. *Cell death discovery*.

[14] Uppala J. K., Gani A. R., Ramaiah K. V. A. (2017). Chemical chaperone, TUDCA unlike PBA, mitigates protein aggregation efficiently and resists ER and non-ER stress induced HepG2 cell death. *Scientific Reports*.

[15] Qu C., Ma J., Liu X. (2017). Dihydroartemisinin exerts anti-tumor activity by inducing mitochondrion and endoplasmic reticulum apoptosis and autophagic cell death in human glioblastoma cells. *Frontiers in Cellular Neuroscience*.

[16] Zinszner H., Kuroda M., Wang X. (1998). CHOP is implicated in programmed cell death in response to impaired function of the endoplasmic reticulum. *Genes & Development*.

[17] Yamaguchi H., Wang H. G. (2004). CHOP is involved in endoplasmic reticulum stress-induced apoptosis by enhancing DR5 expression in human carcinoma cells. *Journal of Biological Chemistry*.

[18] Lu J. J., Chen S. M., Zhang X. W., Ding J., Meng L. H. (2011). The anti-cancer activity of dihydroartemisinin is associated with induction of iron-dependent endoplasmic reticulum stress in colorectal carcinoma HCT116 cells. *Investigational New Drugs*.

[19] Shimizu T., Kamel W. A., Yamaguchi-Iwai S., Fukuchi Y., Muto A., Saya H. (2017). Calcitriol exerts an anti-tumor effect in osteosarcoma by inducing the endoplasmic reticulum stress response. *Cancer Science*.

[20] Shen S., Zhou M., Huang K. (2017). Blocking autophagy enhances the apoptotic effect of 18*β*-glycyrrhetinic acid on human sarcoma cells via endoplasmic reticulum stress and JNK activation. *Cell Death & Disease*.

[21] Daniel V. C., Marchionni L., Hierman J. S. (2009). A primary xenograft model of small-cell lung cancer reveals irreversible changes in gene expression imposed by culture in vitro. *Cancer Research*.

[22] Herrmann D., Conway J. R. W., Vennin C. (2014). Three-dimensional cancer models mimic cell-matrix interactions in the tumour microenvironment. *Carcinogenesis*.

[23] Boj S. F., Hwang C. I., Baker L. A. (2015). Organoid models of human and mouse ductal pancreatic cancer. *Cell*.

[24] Zhang H. C., Kuo C. J. (2015). Personalizing pancreatic cancer organoids with hPSCs. *Nature Medicine*.

[25] Salahudeen A. A., Kuo C. J. (2015). Toward recreating colon cancer in human organoids. *Nature Medicine*.

[26] Liu X., Ory V., Chapman S. (2012). ROCK inhibitor and feeder cells induce the conditional reprogramming of epithelial cells. *The American Journal of Pathology*.

[27] Liu X., Krawczyk E., Suprynowicz F. A. (2017). Conditional reprogramming and long-term expansion of normal and tumor cells from human biospecimens. *Nature Protocols*.

[28] Tang Y., Jacobi A., Vater C., Zou L., Zou X., Stiehler M. (2015). Icariin promotes angiogenic differentiation and prevents oxidative stress-induced autophagy in endothelial progenitor cells. *Stem Cells*.

[29] Tang Y., Jacobi A., Vater C., Zou X., Stiehler M. (2014). Salvianolic acid B protects human endothelial progenitor cells against oxidative stress-mediated dysfunction by modulating Akt/mTOR/4EBP1, p38 MAPK/ATF2, and ERK1/2 signaling pathways. *Biochemical Pharmacology*.

[30] Zhang R., Xia Y., Wang Z. (2017). Serum long non coding RNA MALAT-1 protected by exosomes is up-regulated and promotes cell proliferation and migration in non-small cell lung cancer. *Biochemical and Biophysical Research Communications*.

[31] Huang S., Wa Q., Pan J. (2017). Downregulation of miR-141-3p promotes bone metastasis via activating NF-*κ*B signaling in prostate cancer. *Journal of Experimental & Clinical Cancer Research*.

[32] Choi J. H., Jeong Y. J., Yu A. R. (2017). Fluoxetine induces apoptosis through endoplasmic reticulum stress via mitogen-activated protein kinase activation and histone hyperacetylation in SK-N-BE(2)-M17 human neuroblastoma cells. *Apoptosis*.

[33] Hu X., Christian P. J., Thompson K. E., Glenn Sipes I., Hoyer P. B. (2001). Apoptosis induced in rats by 4-vinylcyclohexene diepoxide is associated with activation of the caspase cascades. *Biology of Reproduction*.

[34] Chen X. T., Cheng C., Xie Y. D. (2019). Pristimerin induces cell death in conditionally reprogrammed patient- derived lung cancer cells by modulating Notch signaling. *Chinese Journal of Hospital Pharmacy*.

[35] Zhang L., Wang Y., Zhang L. (2019). ZBTB7A, a miR-663a target gene, protects osteosarcoma from endoplasmic reticulum stress-induced apoptosis by suppressing LncRNA GAS5 expression. *Cancer Letters*.

[36] Kaur B., Bhat A., Chakraborty R. (2018). Proteomic profile of 4-PBA treated human neuronal cells during ER stress. *Mol Omics*.

[37] Rasheva V. I., Domingos P. M. (2009). Cellular responses to endoplasmic reticulum stress and apoptosis. *Apoptosis : an international journal on programmed cell death*.

[38] Campochiaro P. A., Strauss R. W., Lu L. (2015). Is there excess oxidative stress and damage in eyes of patients with retinitis pigmentosa?. *Antioxidants & Redox Signaling*.

[39] Sinha U. K., Kundra A., Scalia P. (2019). Expression of EphB4 in head and neck squamous cell carcinoma. *Ear, Nose, & Throat Journal*.

[40] Kumar S. R., Masood R., Spannuth W. A. (2007). The receptor tyrosine kinase EphB4 is overexpressed in ovarian cancer, provides survival signals and predicts poor outcome. *British Journal of Cancer*.

[41] Ferguson B. D., Liu R., Rolle C. E. (2013). The EphB4 receptor tyrosine kinase promotes lung cancer growth: a potential novel therapeutic target. *PLoS One*.

[42] Hasina R., Mollberg N., Kawada I. (2013). Critical role for the receptor tyrosine kinase EPHB4 in esophageal cancers. *Cancer Research*.

[43] Zheng M. F., Ji Y., Wu X. B., Ye S. G., Chen J. Y. (2012). EphB4 gene polymorphism and protein expression in non-small-cell lung cancer. *Molecular Medicine Reports*.

[44] Sagar V., Vatapalli R., Lysy B. (2019). EPHB4 inhibition activates ER stress to promote immunogenic cell death of prostate cancer cells. *Cell Death & Disease*.

[45] Guo Y., Zhang W., Yan Y. Y. (2013). Triterpenoid pristimerin induced HepG2 cells apoptosis through ROS-mediated mitochondrial dysfunction. *Journal of BUON*.

[46] Eum D. Y., Byun J. Y., Yoon C. H. (2011). Triterpenoid pristimerin synergizes with taxol to induce cervical cancer cell death through reactive oxygen species-mediated mitochondrial dysfunction. *Anti-Cancer Drugs*.

[47] Zhao H., Wang C., Lu B. (2016). Pristimerin triggers AIF-dependent programmed necrosis in glioma cells via activation of JNK. *Cancer Letters*.

[48] Du Y., Wang K., Fang H. (2006). Coordination of intrinsic, extrinsic, and endoplasmic reticulum-mediated apoptosis by imatinib mesylate combined with arsenic trioxide in chronic myeloid leukemia. *Blood*.

[49] Green D. R., Reed J. C. (1998). Mitochondria and apoptosis. *Science*.

[50] Mukherjee A., Khuda-Bukhsh A. R. (2015). Quercetin down-regulates IL-6/STAT-3 signals to induce mitochondrial-mediated apoptosis in a nonsmall-cell lung-cancer cell line, A549. *Journal of pharmacopuncture*.

[51] Gharabaghi M. A. (2018). Diagnostic investigation of BIRC6 and SIRT1 protein expression level as potential prognostic biomarkers in patients with non-small cell lung cancer. *The Clinical Respiratory Journal*.

[52] Hitomi J., Katayama T., Eguchi Y. (2004). Involvement of caspase-4 in endoplasmic reticulum stress-induced apoptosis and A*β*-induced cell death. *The Journal of Cell Biology*.

[53] Yamamuro A., Kishino T., Ohshima Y. (2011). Caspase-4 directly activates caspase-9 in endoplasmic reticulum stress–induced apoptosis in SH-SY5Y cells. *Journal of Pharmacological Sciences*.

[54] Keestra-Gounder A. M., Byndloss M. X., Seyffert N. (2016). NOD1 and NOD2 signalling links ER stress with inflammation. *Nature*.

[55] Hashimoto S., Ishii A., Kamano N. (2018). Endoplasmic reticulum stress responses in mouse models of Alzheimer’s disease: overexpression paradigm versus knockin paradigm. *The Journal of Biological Chemistry*.

[56] Schonthal A. H. (2013). Pharmacological targeting of endoplasmic reticulum stress signaling in cancer. *Biochemical Pharmacology*.

[57] Bhat T. A., Chaudhary A. K., Kumar S. (2017). Endoplasmic reticulum-mediated unfolded protein response and mitochondrial apoptosis in cancer. *Biochimica et Biophysica Acta*.

[58] Tang Z. H., Chen X., Wang Z. Y. (2016). Induction of C/EBP homologous protein-mediated apoptosis and autophagy by licochalcone A in non-small cell lung cancer cells. *Scientific Reports*.

[59] Zeeshan H., Lee G., Kim H.-R., Chae H.-J. (2016). Endoplasmic reticulum stress and associated ROS. *International Journal of Molecular Sciences*.

[60] Seervi M., Rani A., Sharma A. K., Santhosh Kumar T. R. (2018). ROS mediated ER stress induces Bax-Bak dependent and independent apoptosis in response to thioridazine. *Biomedicine & Pharmacotherapy*.

[61] Jacobson J., Duchen M. R. (2002). Mitochondrial oxidative stress and cell death in astrocytes — requirement for stored Ca^2+^ and sustained opening of the permeability transition pore. *Journal of Cell Science*.

[62] Beer D. G., Kardia S. L. R., Huang C.-C. (2002). Gene-expression profiles predict survival of patients with lung adenocarcinoma. *Nature Medicine*.

[63] Yang N. Y., Pasquale E. B., Owen L. B., Ethell I. M. (2006). The EphB4 receptor-tyrosine kinase promotes the migration of melanoma cells through Rho-mediated actin cytoskeleton reorganization. *The Journal of Biological Chemistry*.

[64] Masood R., Kumar S. R., Sinha U. K. (2006). EphB4 provides survival advantage to squamous cell carcinoma of the head and neck. *International Journal of Cancer*.

[65] Adams R. H., Diella F., Hennig S., Helmbacher F., Deutsch U., Klein R. (2001). The cytoplasmic domain of the ligand ephrinB2 is required for vascular morphogenesis but not cranial neural crest migration. *Cell*.

[66] El-Sibai M., Nalbant P., Pang H. (2007). Cdc42 is required for EGF-stimulated protrusion and motility in MTLn3 carcinoma cells. *Journal of Cell Science*.

[67] Weaver A. M. (2006). Invadopodia: specialized cell structures for cancer invasion. *Clinical & Experimental Metastasis*.

