# Supplementary material for: Pristimerin Exacerbates Cellular Injury in Conditionally Reprogrammed Patient-Derived Lung Adenocarcinoma Cells by Aggravating Mitochondrial Impairment and Endoplasmic Reticulum Stress through EphB4/CDC42/N-WASP Signaling
Source: Oxid Med Cell Longev. 2020 Jul 10;2020:7409853. doi: 10.1155/2020/7409853 (PMC7369684; doi:10.1155/2020/7409853)

1. For ROS assay with EphB4 knockdown, the statistical analysis of the 4 groups was

shown as follows:

1. Control & PRIS:


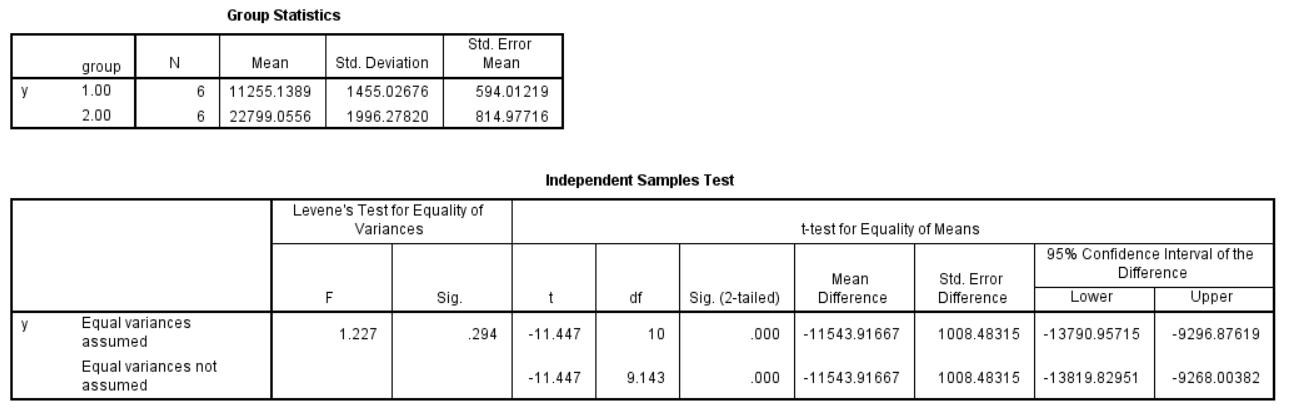


1. Control & EphB4 siRNA:


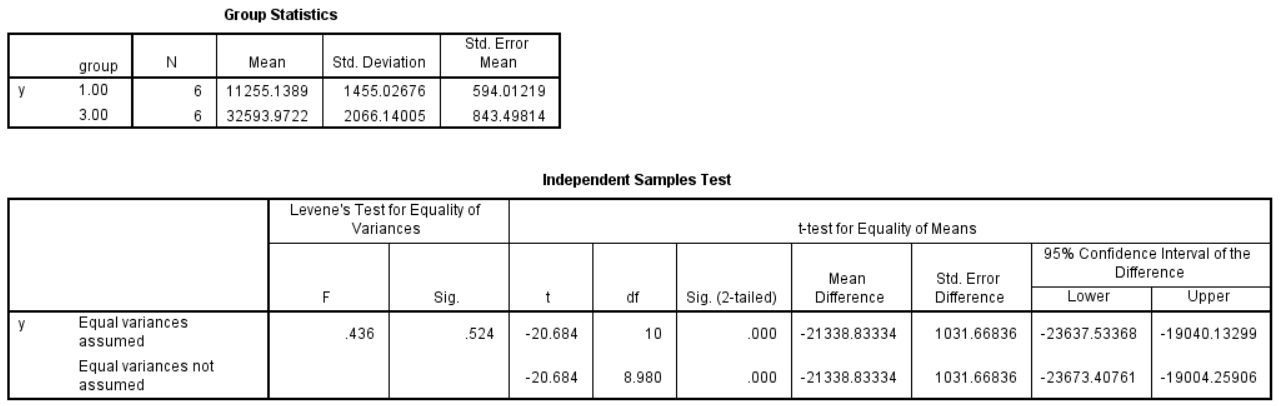


1. Control & PRIS+EphB4 siRNA:


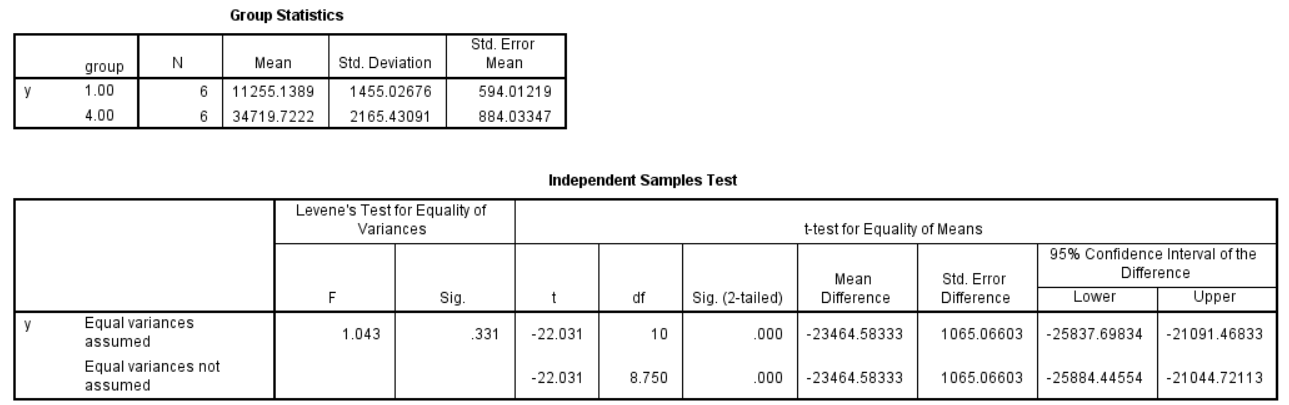


1. EphB4 siRNA & PRIS+EphB4 siRNA:


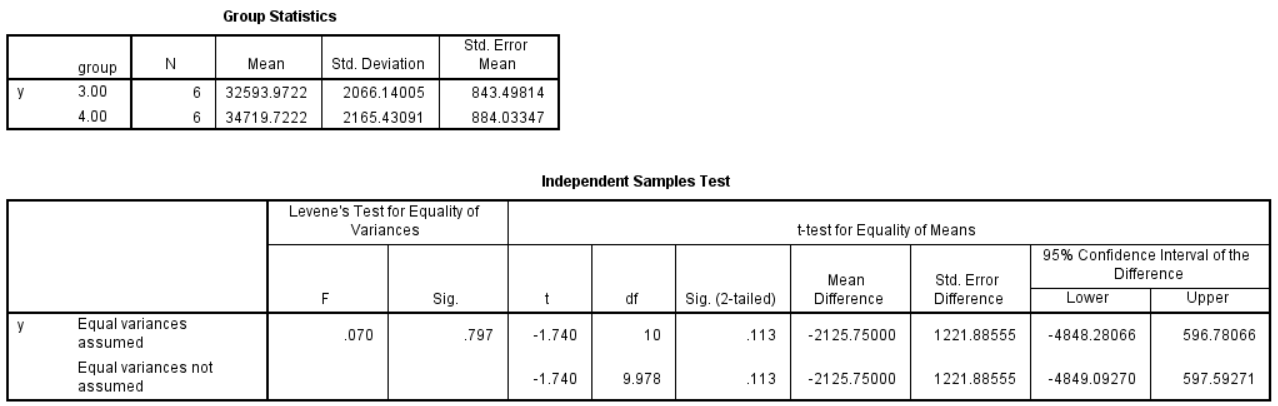


Figure:


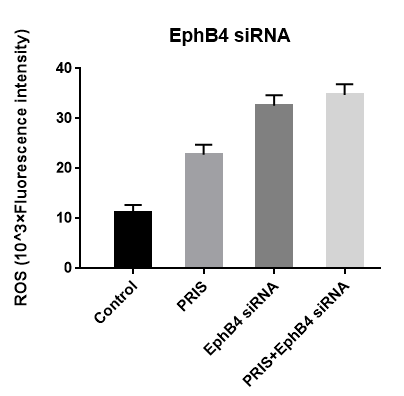


1. For ROS assay with N-WASP knockdown, the statistical analysis of the 4 groups

was shown as follows:

1. Control & PRIS siRNA:


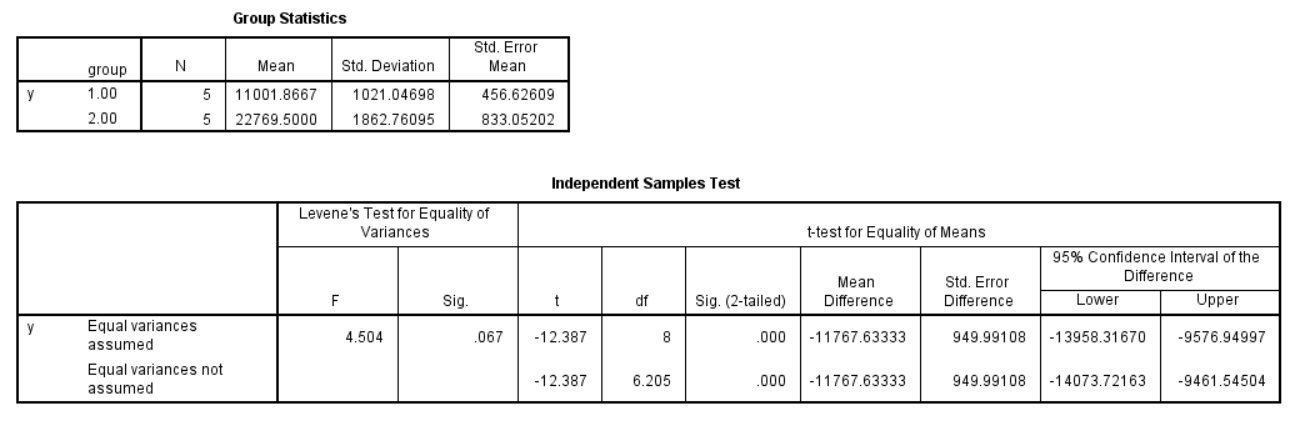


1. Control & N-WASP siRNA:


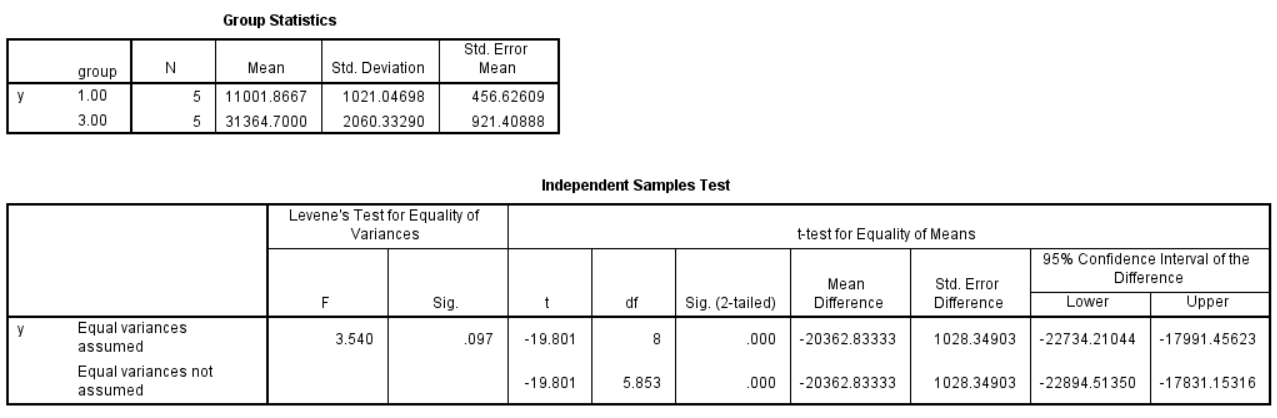


1. Control & PRIS+N-WASP siRNA:


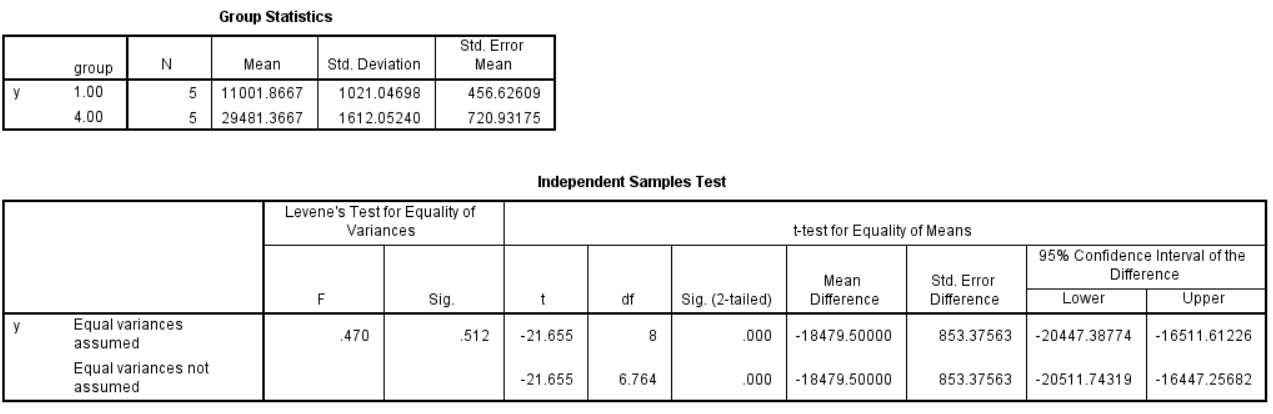


1. N-WASP siRNA & PRIS+N-WASP siRNA:


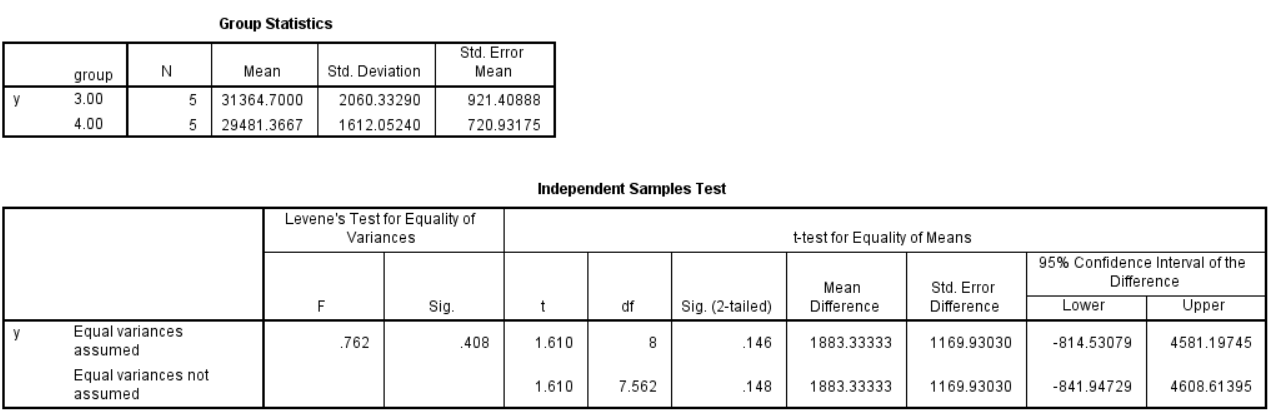


Figure:


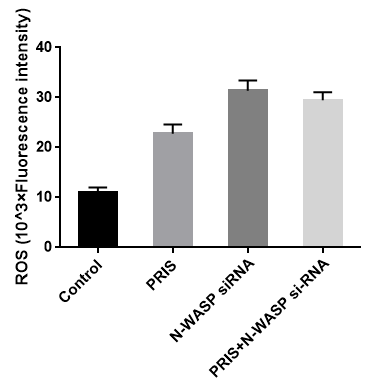

Supplement: Supplementary Materials — This section includes (1) original data.docx, (2) ROS-si-EphB4-original.xlsx, and (3) ROS-si-N-WASP-original.xlsx. [file 7409853.f1.zip › Original data.docx]
